# Understanding the Impact of SAM Fermi Levels on High Efficiency p-i-n Perovskite Solar Cells

**DOI:** 10.1021/acs.jpclett.4c02345

**Published:** 2024-10-16

**Authors:** Fraser J. Angus, Wai Kin Yiu, Hongbo Mo, Tik Lun Leung, Muhammad Umair Ali, Yin Li, Jingbo Wang, Anita. W. Y. Ho-Baillie, Graeme Cooke, Aleksandra B. Djurišić, Pablo Docampo

**Affiliations:** ◧ Department of Chemistry, University of Glasgow, University Avenue, Glasgow G12 8QQ, U.K.; ‡ Department of Physics, The University of Hong Kong, Pokfulam Road, Hong Kong S.A.R, China; § School of Physics, The University of Sydney, Sydney, New South Wales 2006, Australia; ◇ Sydney Nano, The University of Sydney, Sydney, New South Wales 2006, Australia; ∥ Australian Centre for Advanced Photovoltaics (ACAP), School of Photovoltaic and Renewable Energy Engineering, University of New South Wales, Sydney NSW 2052, Australia

## Abstract

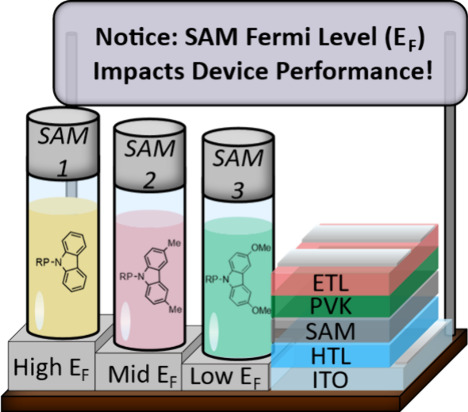

Completing the picture of the underlying physics of perovskite solar cell interfaces that incorporate self-assembled molecular layers (SAMs) will accelerate further progress in p-i-n devices. In this work, we modified the Fermi level of a nickel oxide–perovskite interface by utilizing SAM layers with a range of dipole strengths to establish the link between the resulting shift of the built-in potential of the solar cell and the device parameters. To achieve this, we fabricated a series of high-efficiency perovskite solar cells with no hysteresis and characterized them through stabilize and pulse (SaP), JV curve, and time-resolved photoluminescence (TRPL) measurements. Our results unambiguously show that the potential drop across the perovskite layer (in the range of 0.6–1 V) exceeds the work function difference at the device’s electrodes. These extracted potential drop values directly correlate to work function differences in the adjacent transport layers, thus demonstrating that their Fermi level difference entirely drives the built-in potential in this device configuration. Additionally, we find that selecting a SAM with a deep HOMO level can result in charge accumulation at the interface, leading to reduced current flow. Our findings provide insights into the device physics of p-i-n perovskite solar cells, highlighting the importance of interfacial energetics on device performance.

Perovskite solar cells incorporate a printable family of semiconductors that are low-cost,^1^ highly efficient, and inherently recyclable,^2^ making them a promising candidate for large-scale energy production.^3^ This has generated substantial interest in both academic and industrial research,^4−6^ driving advancements in this technology at an unprecedented pace and already demonstrating performance on par with silicon photovoltaics.^7,8^ Progress was initially fuelled by an increased understanding of the crystallization processes, which enabled the optimization of the perovskite composition.^9,10^ However, the latest leaps in performance have mainly resulted from breakthroughs in the development of interfacial materials and passivation strategies.^8,11^

Interfacial engineering to maximize performance is a common approach in all areas of photovoltaic (PV) research. However, in contrast to other PV systems, the ionic nature of perovskite absorbers^12^ presents a new challenge to overcome and interesting alternative optimization pathways. For example, strategies that incorporate simple ionic compounds to form layered perovskites on the surface of a state-of-the-art perovskite composition,^13^ or simple adsorption on the surface, can effectively passivate surface defects, greatly enhancing device performance.^14^

A recent popular approach in this arena is the inclusion of self-assembled molecular (SAM) layers to improve charge extraction in state-of-the-art solar cells.^15,16^ These molecules form strong dipoles when bound on the surface of typical charge extraction contacts, which leads to shifts in their work function and thus inducing interfacial band bending.^17^ This can result in efficient charge collection, and also make a contact such as indium-doped tin oxide (ITO) charge-selective.^18^ Carbazole-based phosphonic acid derivatives have been shown to lead to particularly high-efficiency devices as a result of surface recombination minimization and excellent energy level alignment.^19^

Despite their widespread success, it is hard to link how a SAM’s dipole, orientation on the surface, and the resulting change in interfacial energetics affect the device parameters. In particular, the power conversion efficiency (PCE) of solar cells including SAMs varies significantly depending on the perovskite composition and device architecture.^20,21^ This results in an extensive trial and error procedure to identify the SAM that provides the optimum device performance. This is in part a result of the inherent uncertainty of energy level diagrams, which are typically put together using a combination of measurements in partial device stacks, for instance, through X-Ray Photoelectron Spectroscopy (XPS), Ultraviolet Photoelectron Spectroscopy(UPS) and electroabsorption (EA) spectroscopy measurements.^22,23^ Furthermore, the typical SAM-based, p-i-n device architecture employs ITO and silver (Ag), whose work functions differ by ∼0.2 eV.^24,25^ In essence, the built-in potential of these devices is almost negligible, yet the devices function extremely efficiently, raising an important question about the driving forces within this device architecture.

We have recently developed a characterization tool based on rapid voltage pulses, termed Stabilize and Pulse (SaP) measurements.^26^ This technique can determine interfacial energy level offsets in complete working devices. The approach takes advantage of the effects of ion accumulation at device interfaces on charge extraction and recombination. This allows the identification of the potential drop which leads to a uniform distribution of ions within the perovskite layer, termed V_flat_.^27^ The developed analysis leads to values with a reasonable uncertainty of around 0.05 V,^26^ for common perovskite compositions and typical ion density, and charge recombination values. Since the flat ion potential of the perovskite (V_flat_) is closely related to the Fermi level positions of the adjacent transport layers,^27^ this measurement can be used to discriminate between small changes (<0.1 eV) in device energetics.^26^ In addition, this tool enables the comparison of device behavior with and without ions as device performance is measured with mobile ions “frozen” in place over a range of applied biases.^27^

In this work, we establish the link between the built-in potential induced by charge extraction layers and the resulting device parameters in high-efficiency perovskite solar cells with no hysteresis. By employing a range of SAMs with increasing dipole strength, we systematically measure the potential across the perovskite layer finding values ranging from 0.6–1.0 V, far above that of the potential difference (V_bi_) of the electrodes. Furthermore, we combine complementary characterization in the form of time-resolved photoluminescence (TRPL) and JV curve analysis. This allows us to discriminate between increased recombination, efficient charge extraction, and potential barriers at the interface. Our results thus provide insights into the device physics of p-i-n perovskite solar cells, highlighting the role of interfacial energetics on device performance.

To explore the influence of the Fermi level shift induced by the SAM layer on perovskite solar cell device behavior, we utilized the following molecules: [2-(9H-carbazol-9-yl)ethyl]phosphonic acid (2PACz), [2-(3,6-dimethoxy-9H-carbazol-9-yl)ethyl]phosphonic acid (MeO-2PACz), and (4-(3,6-dimethyl-9H-carbazol-9-yl)butyl)phosphonic acid (Me-4PACz). These materials have been extensively used in the literature and thus serve as an important benchmark while also delivering high-power conversion efficiencies.^28,29^ Furthermore, the SAMs exhibit a clear trend in dipole strength, going from 2PACz > Me-4PACz > MeO-2PACz,^30,31^ leading to well-differentiated Fermi level shifts upon anchoring on the metal oxide surface, as characterized via UPS/XPS.^32^ These SAMs are thus an ideal starting point for increasing our understanding of device energetics.

We begin by verifying the impact of the SAMs on device performance. Here we focus on a p-i-n configuration using nickel oxide nanoparticles (NiO_*x*_) as the hole transporting layer (HTL). This material has been shown to improve efficiency as well as improve surface coverage compared to bare ITO.^33^ We utilize the archetypal methylammonium lead iodide (MAPI) perovskite as well as an optimized formulation that incorporates a double cation composition of formamidinium and cesium (FA_0.9_Cs_0.1_PbI_2.9_Br_0.1_), abbreviated to “DC” for the remainder of the manuscript. Finally, phenyl-C61-butyric acid methyl ester (PCBM) and bathocuproine (BCP) were used as the electron transport layers (ETL) and Ag as the top electrode. Full fabrication and synthetic procedures for these devices can be found in the Experimental Methods.

To confirm that follow-on comparisons are representative of the device physics and not simply a result of different morphologies upon crystallization, we collected SEM images of all perovskite films, Figures 1a-c (DC) and 1e-g (MAPI), and X -ray diffraction data, Figures 1d (DC) and 1h (MAPI). Both perovskite compositions yield a pure-phase structure with no notable differences between the SAM layer used. The only exception to this is DC on Me-4PACz which resulted in a loss of relative intensity for the peak at 13.8 2θ compared to the other reflections. This indicates that crystallites within this film are more disordered with fewer crystals preferentially oriented in the 110 direction.

**Figure 1:**
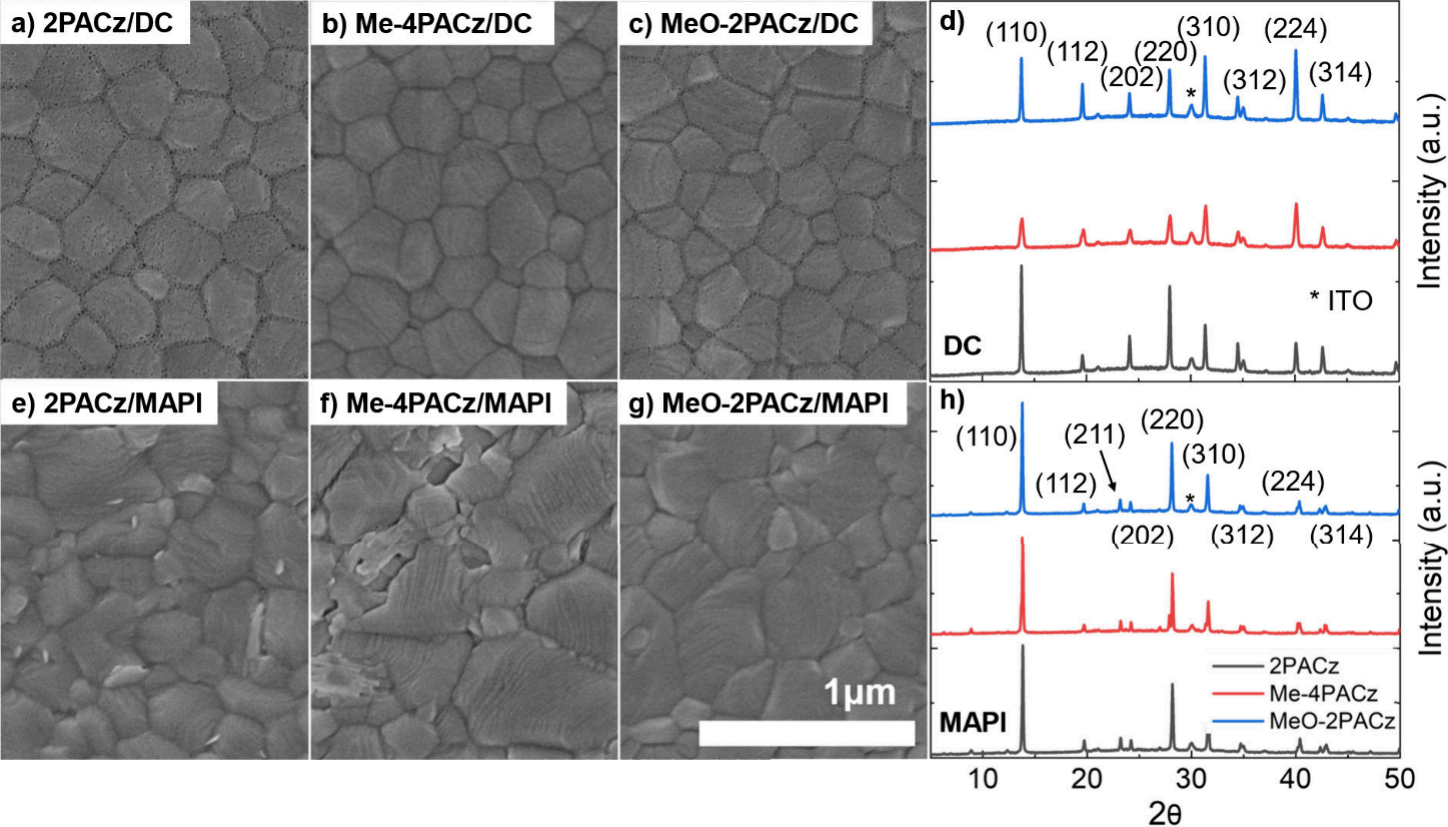
SEM images of DC perovskite on (a) 2PACz, (b) Me-4PACz, and (c) MeO-2PACz. SEM images of MAPI on (e) 2PACz, (f) Me-4PACz, and (g) MeO-2PACz. XRD data for (d) DC and (h) MAPI on all SAMs with 2PACz in black, Me-4PACz in red, and MeO-2PACz in blue.

To quantify the grain size from the SEM images, we selected and measured ∼120 grains for DC, while for the MAPI samples we analyzed ∼70 grains. The mean grain size was calculated by averaging the measured diameters, and the associated error was obtained by calculating the standard deviation of all measured diameters, providing an estimate of the variability in grain distribution. Our results show DC average grain sizes of 274.6 ± 57.5 nm for 2PACz, 253.0 ± 60.6 nm for MeO-2PACz and 258.6 ± 64.6 nm for Me-4PACz. MAPI average grain sizes were consistent for all SAMs with 302.8 ± 81.6 nm for 2PACz, 289.5 ± 104.8 nm for MeO-2PACz and 276.6 ± 100.1 nm for Me-4PACz. As the average grain size differences are smaller than the standard deviation values, we can thus attribute the effects of the SAMs on device performance to changes in interfacial energetics.

The resulting JV curves measured under AM 1.5 solar simulated sunlight for solar cells incorporating the SAM layers are presented in Figures 2a and 2b for both DC and MAPI as the photo absorber layer. We find that the power conversion efficiencies of champion devices exceed 22% for DC and 20% for MAPI. Although solar cells incorporating MAPI lead to some hysteresis in the JV curve under standard scan rate conditions, the DC composition leads to hysteresis-free behavior, as is typical for this material system.^34^ A summary of the champion solar cells for each SAM for both DC and MAPI can be found in the insets of Figure 2a and 2b, respectively. Statistics for the complete series are included in Figure S1 for DC and Figure S2 for MAPI.

**Figure 2:**
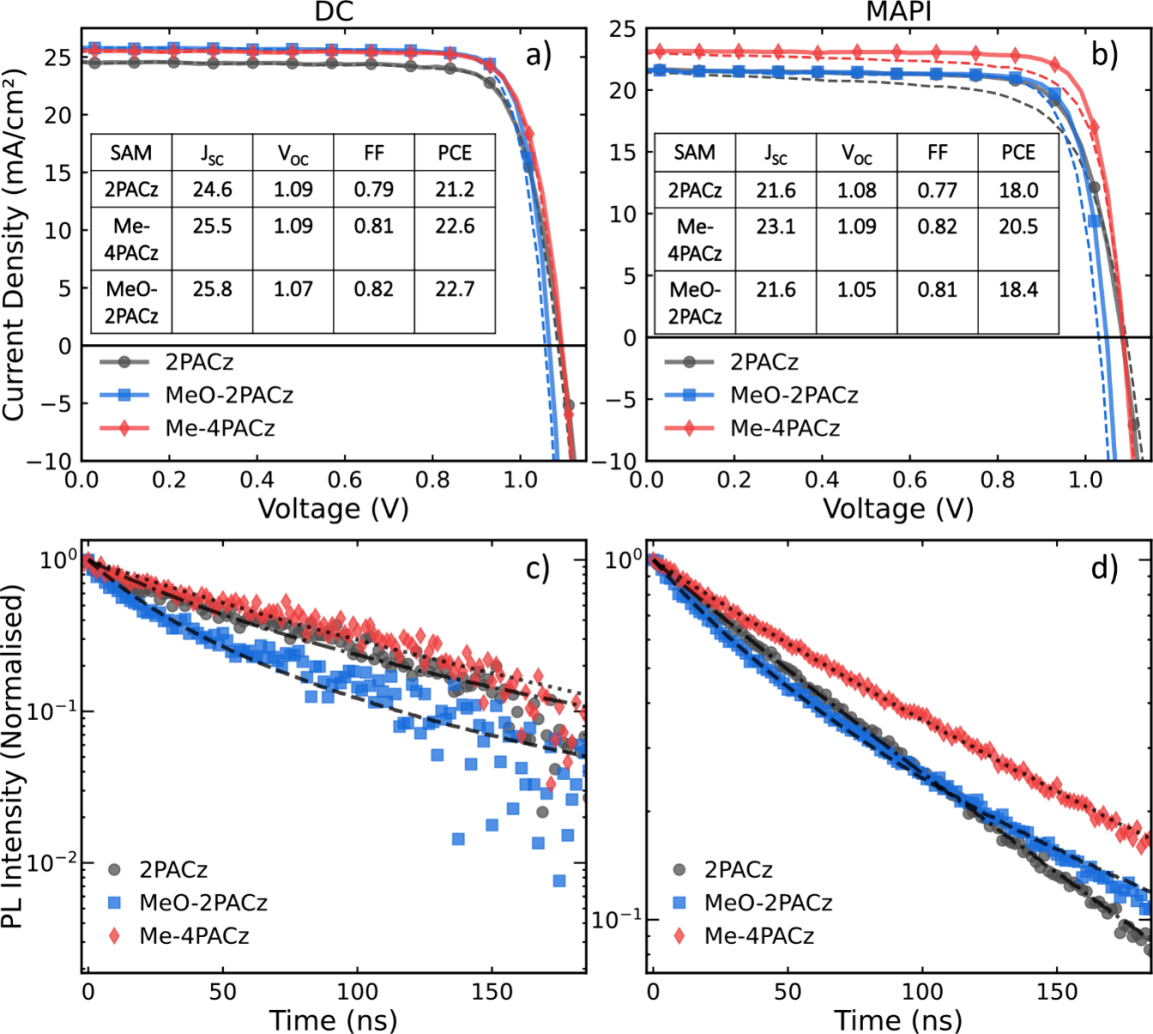
JV curves for champion devices. The forward scans are depicted as dashed lines and the reverse scans as solid lines. The insets contain all the performance parameters for champion devices. Panel (a) shows the JV results for the DC perovskite composition, and panel (b) shows JV curves for the MAPI devices. Normalized Time-resolved photoluminescence (TRPL) decay on an ITO/NiOx/SAM/perovskite partial device stack are shown for (c) DC and (d) MAPI perovskite films. The fits were obtained using the PEARs fitting tool,^35^ and the resulting recombination constants are summarized in Supplementary Tables S1 and S2 for both DC and MAPI.

As can be seen from the JV curves and overall statistics, solar cells incorporating Me-4PACz, on average, yield higher short circuit currents (*J*_SC_) for both MAPI and DC compositions. In contrast, 2PACz-modified NiO_*x*_ interfaces, on average, resulted in lower *J*_SC_ values, while MeO-2PACz yielded intermediate values. Me-4PACz, on average, resulted in higher PCE in comparison to MeO-2PACz and 2PACz across the whole device series. In addition, our results show that using MeO-2PACz led to consistently lower open circuit voltages (*V*_OC_) compared to the other SAMs, while 2PACz and Me-4PACz exhibited very similar *V*_OC_ values across both perovskite compositions. We note here that the *V*_OC_ in these devices is likely limited by PCBM, as is observed more widely in the literature,^36,37^ explaining the small difference in this parameter between DC and MAPI.

To gain insights into the effects of the SAMs on charge extraction and recombination, we performed TRPL measurements on both DC and MAPI, in a multilayer configuration with the ITO/NiO_*x*_/SAM/PVK, as presented in Figures 2c and 2d, respectively. We utilize the PEARs online TRPL fitter that employs a bimolecular-trapping-Auger model and applies a rate equation to extract recombination rates for the different possible recombination processes to analyze the data.^35^ The extracted parameters are displayed in Supplementary Tables 1 and 2 for DC and MAPI compositions, respectively. The results on MAPI films show that the response of the TRPL trace for MeO-2PACz is clearly dominated by bimolecular recombination dynamics (*k*_2_), with the fit returning a value that is nearly an order of magnitude higher than the sample containing Me-4PACz as well as 2PACz. The DC perovskite composition mirrors this trend, with films incorporating MeO-2PACz showing a similar increase in the bimolecular recombination rate (*k*_2_) of over an order of magnitude compared to Me-4PACz.

Counterintuitively, all the TRPL traces exhibit low monomolecular recombination rates, which is also reproduced more widely in the literature. For example, Al-Ashouri et al. show that ITO/2PACz/Perovskite films exhibit decay dynamics similar to those of perovskite films on quartz.^30^ Considering the high efficiency of the resulting solar cells, one would expect *a priori* efficient charge extraction at this interface. Thus, it would be reasonable to assume that the rate corresponding to this process, monomolecular decay dynamics (*k*_1_), should dominate, as is the case for standard HTMs used in perovskite solar cells, e.g. Spiro-OMeTAD or PEDOT:PSS.^38,39^ The TRPL results indicate that, contrary to popular belief, charge extraction using carbazole-derived SAM composite charge extraction layers is not particularly efficient, while also hinting that charge extraction barriers at the interface may be in place.

To further explore the impact of SAM layers on charge extraction and recombination, we obtained KPFM images of samples prepared in the same manner, as shown in Figure S3. Our results show no significant difference in the surface potential of perovskite on MeO-2PACz, whether in the dark or under illumination.^40^ This indicates that photogenerated charge is being lost through recombination. This may be a result of an increased overlap of the photogenerated electron and hole populations, consistent with the TRPL results. In contrast, KPFM images on films containing 2PACz or Me-4PACz show a clear surface potential difference upon illumination; see Figure S3. This indicates that photogenerated charge is more effectively separated, reflecting the band-bending within the device. It is important to note here that measurements performed on half-cells do not necessarily reflect the full device physics. This is particularly true for inverted solar cells using SAMs and 3*D*/2D absorber layers, which can display complex behavior in terms of energy level alignment.^41^

To truly understand the link between energy level alignment and performance parameters, we performed SaP measurements on complete working solar cells.^26,27^ This technique takes advantage of the effects of ion migration within the perovskite layer to extract energy level offsets at the interfaces.^26^ Briefly, a stabilization bias (V_stab_) is applied to the device, and sufficient time is given to allow mobile ionic vacancies^12^ to reach quasi-steady state for that given bias. Then, a rapid voltage pulse is applied and the current is recorded after ∼1 μs, returning the average value for the subsequent 15 μs before returning to the stabilization bias. This way, a JV curve for a given ionic configuration can be reconstructed. The change in gradient of the JV curve around *V*_OC_ is then extracted for each stabilization voltage. The flat ion potential (V_flat_) is identified through a method described previously;^26^ more information on this can be found in the Experimental Methods and Supplementary Note 1. It signifies the point at which the mobile ion distribution is uniform, i.e., there is no excess of ions accumulated at either interface. For an in-depth description of the Stabilize and Pulse measurement see recent work by Hill et al. and Hart et al.^26,27^

The full JV curves and subsequent dJ/dV analysis for all SAM/perovskite combinations obtained through the SaP measurement are found in Supplementary Figures S4 and S5 for MAPI and DC, respectively. To verify that ions do not migrate during the measurement, we reconstructed SaP curves in both forward and reverse scan directions; see Figure S6 for both MAPI and DC where the curves neatly overlap. Additionally, to ensure that the devices are stable during the measurement, we provide the current density over time plots for all devices, as shown in Figures S7 and S8 for MAPI and DC. For clarity, we also include the Current (J) minus the mean Current (J_mean_) over the final 30 s before pulsing. This is shown in Figures S7 and S8 for MAPI and DC. These curves result in values near zero, indicating that no ionic movement occurred during this time.

The extracted gradient around *V*_OC_ for each SAM/perovskite combination is plotted against its stabilization bias, as presented in Figure 3. The gradient value is normalized to allow all curves to be presented in a single figure, allowing for direct comparisons to be made. A summary of the extracted values is presented in Table 1. Our results show a clear trend of the potential drops across the perovskite layer that correlates with the strength of the dipole moment of the underlying SAM layer. We confirm this trend by using a chlorine-substituted carbazole SAM, Cl-2PACz, with a dipole moment of ∼4.6 D,^42,43^ much greater than that of the other materials utilized. With the large dipole induced by Cl-2PACz, we obtain a V_flat_ of >1.20 V for both MAPI and DC devices. This result confirms the correlation between a large V_flat_ and increased dipole strength. The full SaP and dJ/dV analysis for these devices is presented in Supplementary Figure S9.

**Figure 3:**
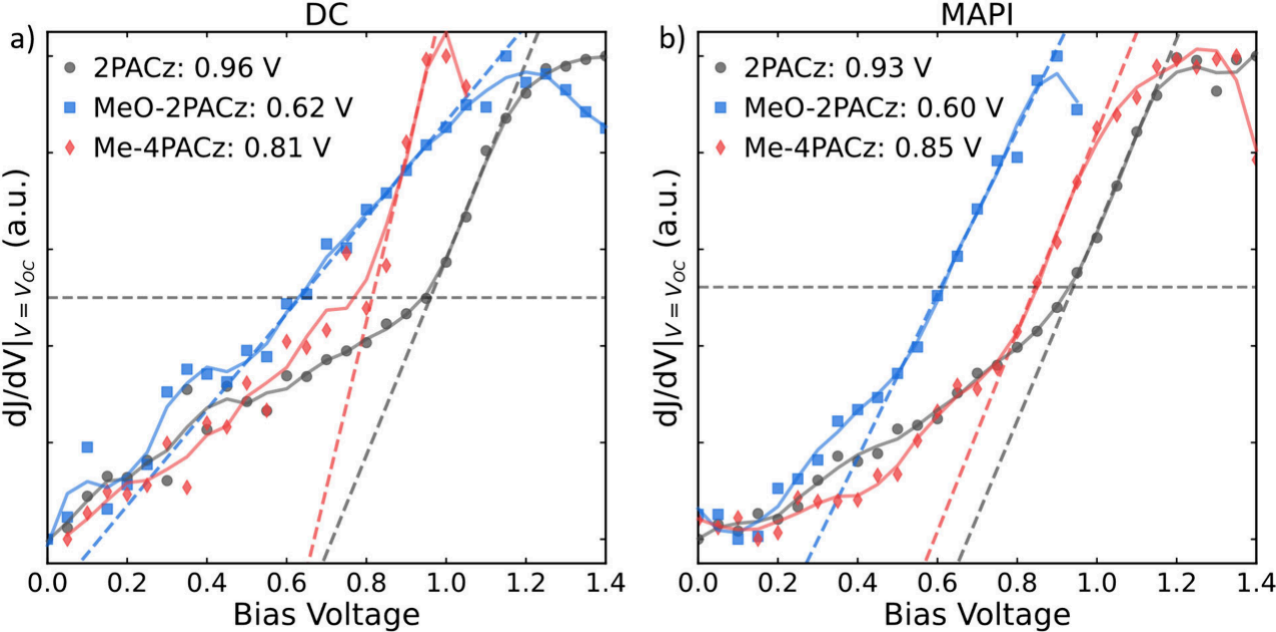
Normalized dJ/dV analysis of devices using all SAM layers for (a) MAPI and (b) DC. 2PACz is in black, Me-4PACz in red, and MeO-2PACz in blue. As a guide to the eye, we include a Savitzky–Golay smoothed line in a lighter shade. The dashed line represents the best linear fit to the steepest part of each curve. The gray horizontal line represents the halfway point of the data and is used in combination with the linear fit to extract the flat ion potential (V_flat_). For more information see Supplementary Note 1.

**Table 1:** Summary Containing Self-Assembled Molecular Layers (SAMs) Utilized, Dipole Moment Extracted from Literature DFT Studies, and the Extracted Flat Ion Potential (V_flat_) of the Perovskite for the Different SAM Layers on MAPI and DC

SAM	Dipole Moment	DC (V_flat_)	MAPI (V_flat_)
2PACz	∼2 D^30^	0.96 ± 0.05 V	0.93 ± 0.05 V
Me-4PACz	∼1.5 D^31^	0.81 ± 0.05 V	0.85 ± 0.05 V
MeO-2PACz	∼0.2 D^30^	0.62 ± 0.05 V	0.60 ± 0.05 V

Overall, these results indicate that the built-in potential of this type of device is entirely driven by the Fermi level positions of the adjacent charge extraction layers rather than the difference in the work function of the electrodes, which is approximately 0.2 eV for this system. This is a typical signature of using doped charge extraction layers,^27^ which is somewhat unexpected since no chemical dopants were added to PCBM in our fabrication protocol, leading us to expect it to be intrinsic *a priori*. However, it has been reported that exposure to air can lead to n-doping of PCBM.^44,45^ Our fabrication protocol includes the transfer of samples from the glovebox to the evaporator in air. We have verified that this exposure leads to a substantial increase in the conductivity of PCBM measured across a patterned ITO substrate, from 6.75 × 10^–8^ S cm^–1^ to 1.97 × 10^–6^ S cm^–1^, as can be seen in Figure S10.

The dJ/dV analysis, derived from the SaP measurements, provides insights into the effects of ion accumulation at interfaces on charge extraction and recombination. The slope of the dJ/dV curve reflects the interplay between ion-modulated interfacial charge recombination and bulk losses. As bulk recombination increases, the interface becomes less dominant, resulting in a less pronounced slope in the dJ/dV curve. In systems with high densities of mobile ions, such as MAPI, interfacial recombination dominates due to the large density of mobile ions, which is typically in the range of 10^17^ to 10^19^ cm^–3^.^46−49^ This results in the well-known JV hysteresis observed in most MAPI-based solar cells regardless of the contact materials used. In contrast, DC perovskites, with fewer and slower-diffusing ionic species,^46^ exhibit reduced interfacial recombination and JV hysteresis. Therefore they are expected to show a shallower slope in the dJ/dV analysis.

This is reflected in the results, as the extracted data shows that the dJ/dV fits for the MAPI devices are more accurate, while the DC perovskite produces a noisier response. Additionally, we note that the DC device incorporating MeO-2PACz has a shallower slope for dJ/dV, as its trace crosses over the other measured devices. This shallow slope indicates reduced ionic impact and, consequently, an increased bulk-to-surface recombination ratio. This finding aligns with our analysis of the TRPL and KPFM data, which suggested an increased overlap of photogenerated charge.

We also note the strong agreement between the values obtained for the different perovskite compositions utilizing the same SAM layers. This consistency is expected, as both perovskite compositions exhibit similar band gaps, evident from our absorption and Tauc analyses of both the DC perovskite in Figures S11 and S12 and for MAPI in Figures S13 and S14. Therefore, it is reasonable to assume that they will exhibit similar valence and conduction band positions, as well as Fermi levels. Furthermore, considering both sets of results, we can be confident that the extracted V_flat_ value is solely a result of the differences in Fermi levels induced by the various SAMs anchored on the metal oxide layer.

To verify that the results extracted from the SaP measurements are reasonable, we compare the V_flat_ values with expected energetic offsets. We reference values obtained by Siekmann et al., who conducted XPS/UPS analyses of the same SAM layers used herein.^32^ In this work, they identified that 2PACz induces the deepest Fermi level shift at ∼ −5.2 eV followed by Me-4PACz at ∼ −5.0 eV and finally MeO-2PACz at ∼ −4.8 eV. The trend also correlates with the strength of the dipole as expected, with a stronger dipole leading to a shift of the Fermi level to deeper values.

Using a value for doped PCBM which is consistently reported around −4.2 eV in the literature,^44,50^ we obtain a conservative yet reasonable estimate of the difference in Fermi levels between the charge extraction layers: approximately 1 eV for 2PACz, 0.80 eV for Me-4PACz and 0.60 eV for MeO-2PACz. These values are in excellent agreement with our extracted V_flat_ values of 0.93, 0.85, and 0.60 ± 0.05 V respectively for MAPI and very similar results for the DC perovskite. This remarkable agreement between measurements highlights the robustness of this measurement technique. Taken together, we clearly show that SaP measurements can be used to accurately determine interfacial energetic modifications made even in highly efficient perovskite solar cells with reduced mobile ion densities and no hysteresis.

With these results in hand, we can now correlate the effect of the Fermi level of the anchored SAM on the metal oxide with the performance of the devices. First, devices incorporating MeO-2PACz result in a clear loss in *V*_OC_ and *J*_SC_ compared to the other studied molecules on average, see Figures S1 and S2. Notably, the loss in *J*_SC_ is an unintuitive result based on the energetic picture of the solar cell as we show in Figure 4. Conventional wisdom would anticipate that this device configuration would lead to the highest *J*_SC_ as a result of the offset at the HTL/Perovskite interface which, in principle, should provide an additional driving force for charge extraction.^51^

**Figure 4:**
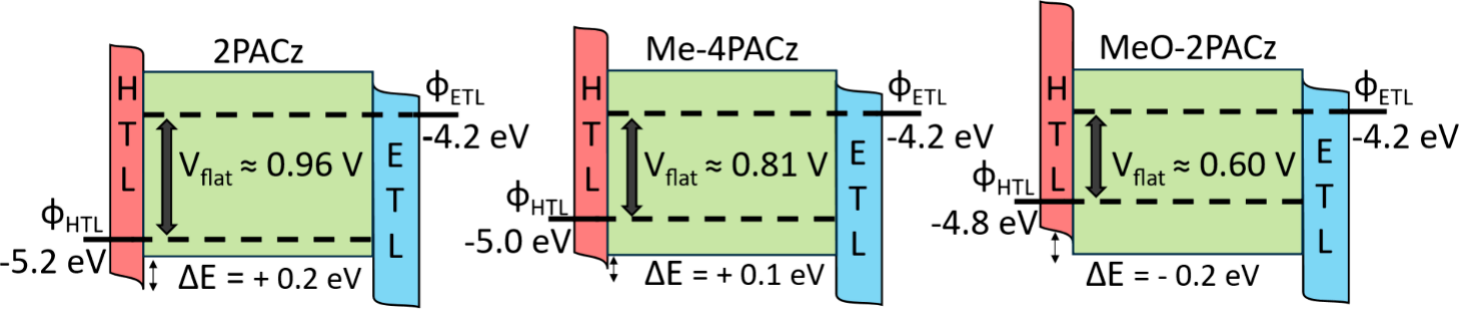
Sketch of reconstructed energy level diagrams for solar cells incorporating each SAM layer. Here the HTL represents the NiO_*x*_/SAM interface and ETL represents the doped PCBM. We place the perovskite valence band edge around −5.4 eV based on literature data.^32^ The electrostatic potential drop across the perovskite layer extracted from the SaP measurements is labeled as V_flat_. The energetic offset from the valence band of the perovskite to the VB of the HTL is labeled Δ*E* and is estimated from values obtained from the literature.^32^

However, we can understand this result in the context of the built-in potential driven by the adjacent charge transport layers, which is approximately equal to V_flat_ for this particular device configuration. Our previous work shows that in “high-injection limited” cases—where interfacial recombination is not the limiting factor—if the built-in potential of the cell is reduced, this will lead to a loss in both *J*_SC_ and *V*_OC_.^27^ This occurs because ionic field screening leads to a bigger overlap of the electron and hole populations, which triggers increased bulk recombination rates. Considering that MeO-2PACz yields a significantly lower V_flat_, this is aligns with the data obtained through TRPL, which indicated that this SAM layer induced a greater bimolecular recombination rate compared to the other SAM layers, as well as the KPFM results, which showed no surface potential difference before and after illumination.

Second, the device utilizing 2PACz showed the largest V_flat_ value, approaching 1 V, due to its strong dipole moment. This results in an increase in *V*_OC_ but a decrease in *J*_SC_ compared to the MeO-2PACz device, a trend also observed in other reports.^30^ Based on our proposed energy level diagrams presented in Figure 4, we can thus assign this loss in current to the formation of a small energetic barrier at the interface, as now the valence band (VB) of the SAM-modified NiO_*x*_ interface is expected to be deeper than the VB of the perovskite. This would, in principle, lead to increased hole accumulation at that interface and thus increased recombination kinetics.

Finally, Me-4PACz-based devices combine the highest current and voltage of the device series. We can now understand that this stems from better alignment of the VB SAM-modified NiO_*x*_ interface to the VB of the perovskite which exhibits an estimated small offset of approximately 0.1 eV. This minimizes charge accumulation at that interface and thus recombination compared to 2PACz, while still maintaining a high enough built-in potential to sustain the high voltages possible for these systems. This, again, is in agreement with the TRPL measurement where we observed the slowest decay kinetics. Our KPFM results showed a build-up of charge at the exposed perovskite surface after illumination, consistent with the existence of a (small) potential barrier for charge extraction at the SAM/perovskite interface.

However, the device results show that this potential barrier does not impact performance. Similar observations were provided by Xu et al. who utilized an electron transport layer that presented a slight barrier to charge extraction and were capable of obtaining high efficiencies.^52^ In fact, a small energetic offset at the interface may be beneficial to boost the open circuit voltage, as observed by Chen et al. for devices incorporating a series of layered perovskites as interface modifiers.^53^ This is consistent with the increased V_OC_ observed for devices incorporating 2PACz and Me-4PACz used herein.

In summary, using a novel stabilize and pulse measurement technique, we have examined the impact of SAM-induced Fermi-level position in high-efficiency p-i-n perovskite solar cells. This technique allowed us to directly measure the flat ion potential (V_flat_) across two perovskite compositions, one based on FA and Cs as well as MAPI. We extract this parameter for solar cells incorporating MeO-2PACz, Me-4PACz, and 2PACz yielding approximate values of 0.60, 0.80, and 0.95 V respectively, with similar values for both perovskite compositions. Our results clearly show that a low V_flat_ consistently led to losses in *J*_SC_ and *V*_OC_. Furthermore, we show that increasing V_flat_ to the middle value of 0.80 V leads to significant improvements in device performance. However, further increases to V_flat_ yield no improvement in performance as the stronger dipole of 2PACz likely pulls the NiO_*x*_/SAM VB position below that of the perovskite, forming an interfacial energy barrier to charge extraction. This leads to charge collection losses, reducing the overall performance of the system. We confirm this hypothesis through KPFM and TRPL measurements. KPFM highlights that in the case of a large V_flat_, there is a significant difference in surface potential in the dark and under illumination, indicating the presence of an energetic barrier. TRPL measurements show particularly low monomolecular recombination constants, indicating poor charge extraction rates, which further supports the existence of potential barriers at this interface. Our results go a long way to explain why different SAMs yield optimum device performance depending on the device architecture. In particular, our results indicate that to fully capitalize on the large built-in potentials induced by 2PACz, a bromide-rich perovskite composition may be optimal to prevent the formation of an energetic barrier by deepening the perovskite’s valence band. In contrast, researchers seeking to optimize low-bandgap perovskites will likely find that MeO-2PACz, and its associated lower V_flat_, will lead to performance gains. Overall, this work demonstrates the importance of fully characterizing the material systems to select the SAM that will enable the highest performance gains.

## Experimental Methods

### NiOx Synthesis

Six g of NiNO_3_·6H_2_O was dissolved in 80 mL of deionized (DI) water with stirring until a clear solution was obtained. Then 80 mL of NaOH (1 mol/L in DI water) was dripped into a nitrate solution, controlling the speed as continuous droplets. The final solution was kept stirring for 5 min. Afterward, the light green product was collected via centrifugation at 10,000 rpm after washing with DI water several times. The product was then freeze-dried for at least 48 h and annealed at 270 °C for 2 h to obtain black NiO_x_ nanoparticles powder.

### p-i-n Perovskite Solar Cell Fabrication

The patterned ITO substrates were cleaned by detergent (Decon 90, 1% in DI water), DI water, acetone and ethanol by sonication for 15 min in each solvent sequentially. The washed substrates were blow-dried with N_2_ and treated with oxygen plasma at 10 V for 10 s. Twenty mg/mL in DI water of NiO_x_ nanoparticles was spin-coated onto the ITO substrate at 4000 rpm for 30 s, followed by annealing at 110 °C for 10 min. Samples with coated NiO_x_ were transferred to a glovebox. 0.5 mg/mL in IPA of SAMs (2PACz, Me-4PACz and MeO-2PACz) was spin-coated at 4000 rpm for 30 s and annealed at 100 °C for 10 min. The MAPI solution was composed of 750 mg of PbI_2_ and 240 mg of MAI in 1 mL of DMF/DMSO (4:1), and the DC solution was composed of 433 mg PbI_2_, 155 mg of FAI, 26 mg of CsI and 22 mg PbBr in 571 μL of DMF and 143 μL of DMSO. Both perovskite precursor solutions were heated overnight at 60 °C and filtered before use. MAPI perovskite solution was spin-coated by two-step processes at 1000 rpm for 5 s and 5000 rpm for 25 s. At 5 s after the start of the first step, 50 μL of MAPI perovskite solution was dropped onto the substrate and at 5 s after the start of the second step, 300 μL of chlorobenzene (CB) antisolvent was dropped onto the MAPI perovskite film.

DC perovskite solution was dispensed on the substrate, followed by two-step processes at 2000 rpm for 10 s and 4000 rpm for 30 s. During the final 10 s of the 2-step spin-coating, 250 μL of CB antisolvent was dropped onto the DC perovskite film. Both perovskite films were annealed at 100 °C for 30 min. To obtain a layered perovskite film, 1 mg/mL in isopropanol (IPA) of PEAI solution was spin-coated on the top of the DC perovskite layer at 5000 rpm for 30 s, followed by annealing at 100 °C for 3 min. Twenty mg/mL in CB of PCBM solution was spin-coated at 1200 rpm for 30 s, followed by annealing at 100 °C for 10 min. 0.5 mg/mL in IPA of BCP was spin-coated at 4000 rpm for 30 s without annealing. Finally, a 100 nm Ag cathode was evaporated through a shadow mask to obtain a device with an electrode area of 0.09 cm^2^. Perovskite solar cells were encapsulated by a microscope glass with polyisobutylene (PIB) tape and edge sealing by epoxy curving with UV.

### Characterization

XRD patterns were measured using a Rigaku MiniFlex600 with Cu Kα X-ray source in θ–2θ scan mode with steps of 0.01° from 5° to 50°. SEM was measured using a Hitachi S-4800, and the acceleration voltage used was 5 kV. The excitation source for TRPL measurement was a 375 nm 200 ns laser diode (Edinburgh FLS1000 Photoluminescence Spectrometer) with switchable repetition rates operating at 1 MHz and 200 kHz. All samples had the structure ITO/NiOx NPs/SAMs/PVK, prepared by the spin coating method, under the same conditions as the solar cell devices. UV–vis absorption measurements were carried out using a Shimadzu UV–vis-NIR spectrophotometer, UV-3600.

Conductivity measurement was calculated by analyzing the current–voltage characteristics of PCBM film (PCBM dissolved in CB on the pattern ITO substrates by spin coating method). The direct current conductivity of thin film (σ) with units of Siemens per centimeter (S/cm) could be measured by σ = 1/ρ = (*l*)/(*A*·*R*) where ρ, *l*, *A* and *R* are resistivity, length of the thin film, the cross-sectional area of the thin film and resistance of given thin film, respectively. J-V measurements were performed using a Keithley 2400 source measure unit under 1 sun illumination with 100 mW/cm^2^ intensity and AM 1.5G spectrum (ABET Sun 2000 solar simulator with calibration by Enli PVM silicon standard reference cell). All devices were measured after encapsulation in ambient conditions (room temperature, RH 60–70%) using an aperture mask of 0.04 cm^2^. The I–V was scanned with a step voltage of 0.01 V and a 10 ms delay. The reverse scan was performed from 1.2 V to −0.2 V, and the forward scan was performed from −0.2 to 1.2 V.

Atomic force microscopy (AFM) and Kelvin probe force microscopy (KPFM) images of different samples were obtained from a Neaspec s-SNOM system with a PtIr_5_ coated AFM probe (Arror EFM, Nanoworld). Illumination on the sample was done with the lighting LED in the sample compartment. The work function (WF) of the sample was obtained by subtracting the surface potential (SP) of a sample from the tip’s work function, which was calibrated with a gold film. All samples were prepared with the structure of ITO/NiOx NPs/SAMs/DC/PEAI using the spin coating, under the same conditions as the solar cell devices.

Stabilize and Pulse measurements were conducted on a home-built setup. Measurements were performed on an Ossila Source Meter Unit. The source delay was set to 1 μs with the extracted value being an average of the following 15 μs. A Cree High Power white LED was used as the light source. Devices were measured on the solar simulator as previously described before being used in the SaP rig. The light intensity was then calibrated to provide the same output current as obtained during the solar simulator measurement. 50 mV step voltages were used for each device to prevent degradation due to the lengthy time scales of this measurement. A stabilization of 120 s was sufficient for the device to provide a stable current output. In the main text, the stabilization bias reported is up to the necessary bias required before a drop in the gradient is observed. We note however that the devices were still stable at these high voltage biases as can be seen in the Supporting Information.

To extract the electrostatic potential drop across the perovskite for each device first 5–7 points were taken around open circuit voltage and a third order polynomial fit, the gradient was analyzed giving dJ/dV. A 7-point window third-order polynomial Savitzky–Golay smoothing filter was then applied to this data and the second derivative (d^2^J/dV^2^) of the smoothed line was used to obtain the inflection point or steepest gradient. From this obtained point a range of points above and below this were used to fit multiple linear fits through the steepest section of the data to find where the linear fit crosses the midpoint of the maxima and minima of the sigmoid. We note that the minima is an average of the first two points weighted 80% to the first point as this provides a more representative look at the data. Multiple linear fits are then made through the range of points to obtain a range of V_flat_ values. A weighted average calculation, described in Supplementary Note 1, is then used from these points to provide the V_flat_ value.

## Supporting Information Available

Parameters extracted from rate equation used to characterize TRPL data; device performance parameters; KPFM imaging of all materials; Stabilize and Pulse measurement and V_flat_ analysis of all devices; analysis of Stabilize and Pulse measurements forward and reverse scans; analysis of Stabilize and Pulse measurement device stability; Stabilize and Pulse measurement of additional SAM layer; conductivity measurement of extraction layer; UV absorption spectra and Tauc analysis of perovskites; supplementary note describing Stabilize and Pulse analysis methodology (PDF)
Transparent Peer Review report available (PDF)
