# Supplementary material for: Understanding the Impact of SAM Fermi Levels on High Efficiency p-i-n Perovskite Solar Cells
Source: J Phys Chem Lett. 2024 Oct 16;15(42):10686–95. doi: 10.1021/acs.jpclett.4c02345 (PMC11514011; doi:10.1021/acs.jpclett.4c02345)
Supplement: Supplementary file 1 — jz4c02345_si_001.pdf [file jz4c02345_si_001.pdf]

Supplementary Information for: Understanding the Impact of SAM Fermi Levels on High Efficiency p-i-n Perovskite Solar Cells

Fraser J. Angus[1,†], Wai Kin Yiu[1,†], Hongbo Mo[2], Tik Lun Leung[3], Muhammad Umair Ali[2], Yin Li[2], Jingbo Wang[2], Anita. W. Y. Ho-Baillie[3,4], Graeme Cooke[1,], Aleksandra B. Djurišić[2,*], Pablo Docampo[1,*]*

[1] Department of Chemistry, University of Glasgow, University Avenue, Glasgow, U.K.

[2] Department of Physics, The University of Hong Kong, Pokfulam Road, Hong Kong S.A.R, China

[3] School of Physics, The University of Sydney, Sydney, New South Wales 2006, Australia; Sydney Nano, The University of Sydney, Sydney, New South Wales 2006, Australia

[4] Australian Centre for Advanced Photovoltaics (ACAP), School of Photovoltaic and Renewable Energy Engineering, University of New South Wales, Sydney NSW 2052, Australia

* pablo.docampo@glasgow.ac.uk; dalek@hku.hk; graeme.cooke@glasgow.ac.uk

Supplementary Tables

For the TRPL model fitting, the charge density following excitation by the laser pulse was estimated based on a reasonable value for the fluence that yields a bimolecular recombination rate consistent with reported literature values.[1] However, considering the same laser with the same pulse settings was used on all the perovskite films fabricated with the same protocol, it is reasonable to assume that the same initial charge density will be generated for all studied samples. Therefore, although the absolute number recorded is unlikely to be completely accurate, the analysis should hold for relative comparisons between the different SAM layers used.

$$\frac{dn}{dt} = -k_1 n(t) - k_2 n^2(t)$$

SAM	k_1 (ns^{-1})	k_1 (%)	$1/k_1$ (ns)	k_2 (cm^3ns^{-1})	k_2 (%)	r^2
2PACz	$1.04x10^{-3}$	14.67	961.5	$1.80x10^{-18}$	85.33	0.9402
MeO-2PACz	**Fixed*:** $5x10^{-4}$	4.36	2000 (fixed)	$3.58x10^{-18}$	95.64	0.9551
Me-4PACz	$3.13x10^{-3}$	52.51	319.5	$2x10^{-19}$	47.49	0.9488

Table S1. Extracted TRPL rate values for DC using the rate equation for the bimolecular-trapping model, obtained using the PEARs fitting tool. Values for 2PACz, MeO-2PACz and Me-4PACz are shown with the partial device stack ITO/NiOx/SAM/perovskite being used. The rate equation is shown above the table where k_1 is the rate of monomolecular recombination, k_2 is the rate of bimolecular recombination, n is the photoexcited carrier concentration and t is time. *The value for k_1 for MeO-2PACz was fixed to provide a non-zero number whilst retaining a reasonable fit to the data.

$$\frac{dn}{dt} = -k_1 n(t) - k_2 n^2(t)$$

SAM	k_1 (ns^{-1})	k_1 (%)	$1/k_1$ (ns)	k_2 ($cm^3 ns^{-1}$)	k_2 (%)	r^2
2PACz	5.66×10^{-3}	83.26	176.7	3.24×10^{-19}	16.74	0.9994
MeO-2PACz	2.87×10^{-4}	4.22	3484.32	1.93×10^{-18}	95.78	0.9985
Me-4PACz	3.37×10^{-3}	67.12	296.7	4.51×10^{-19}	32.89	0.9989

Table S1. Extracted TRPL rate values for MAPI using the rate equation for the bimolecular-trapping model, obtained using the PEARs fitting tool. Values for 2PACz, MeO-2PACz and Me-4PACz are shown with the partial device stack ITO/NiOx/SAM/perovskite being used. The rate equation is shown above the table where k_1 is the rate of monomolecular recombination, k_2 is the rate of bimolecular recombination, n is the photoexcited carrier concentration and t is time.

Supplementary Figures

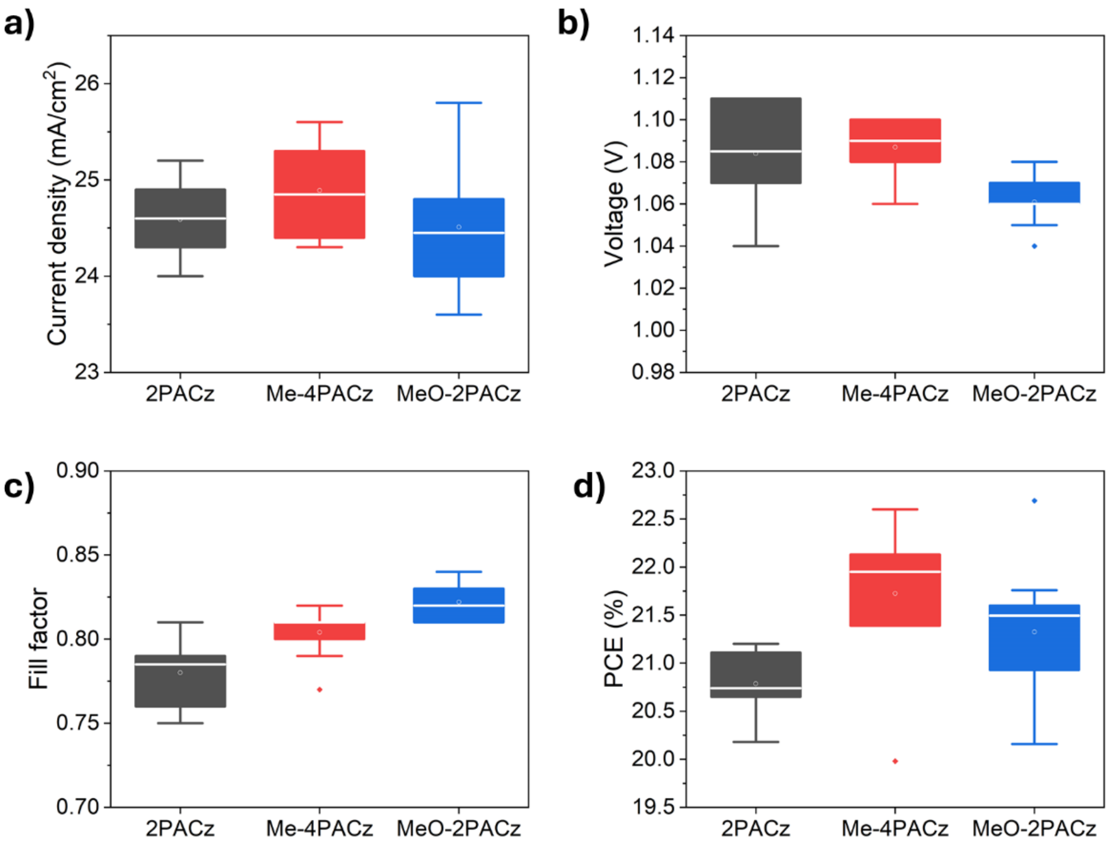

Figure S1. Performance parameters of DC PSCs for different SAMs under simulated solar illumination for forward scan; a) J_{SC}; b) V_{OC}; c) FF and d) PCE. (10 devices for each condition).

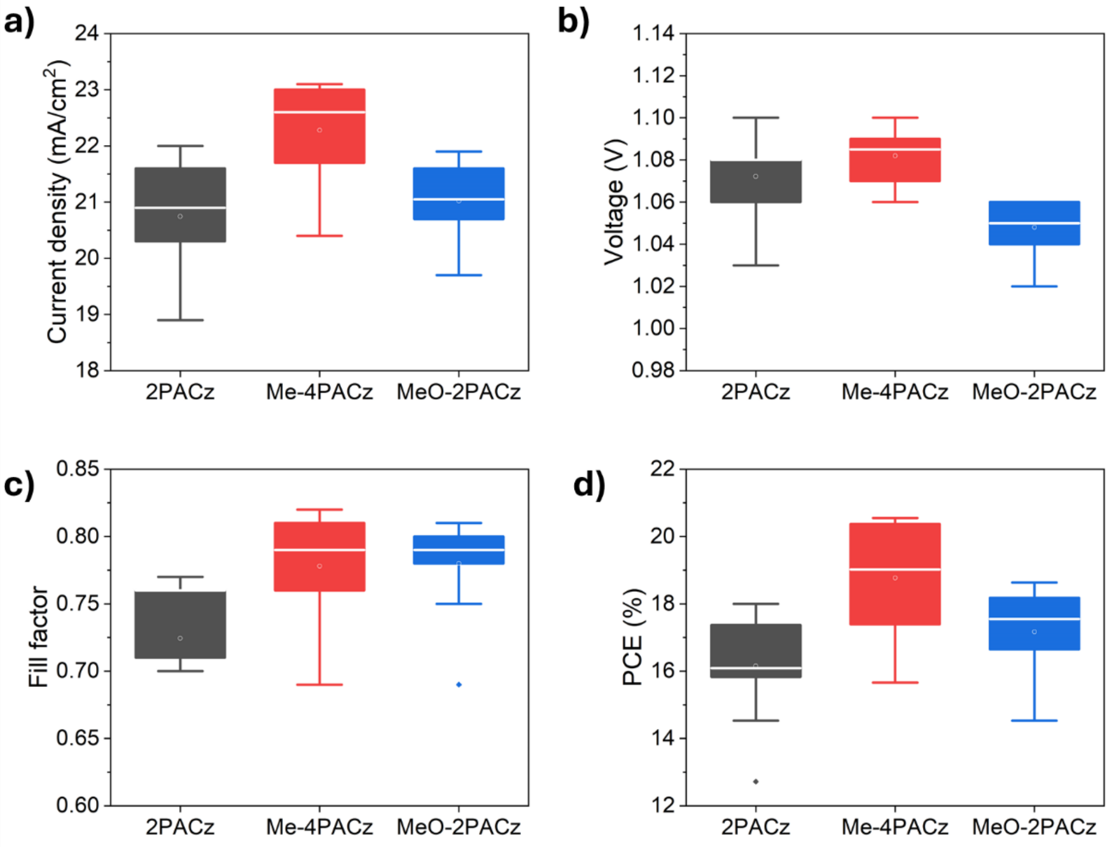

Figure S2. Performance parameters of MAPI PSCs for different SAMs under simulated solar illumination for forward scan; a) Jsc; b) Voc; c) FF and d) PCE. (10 devices for each condition)

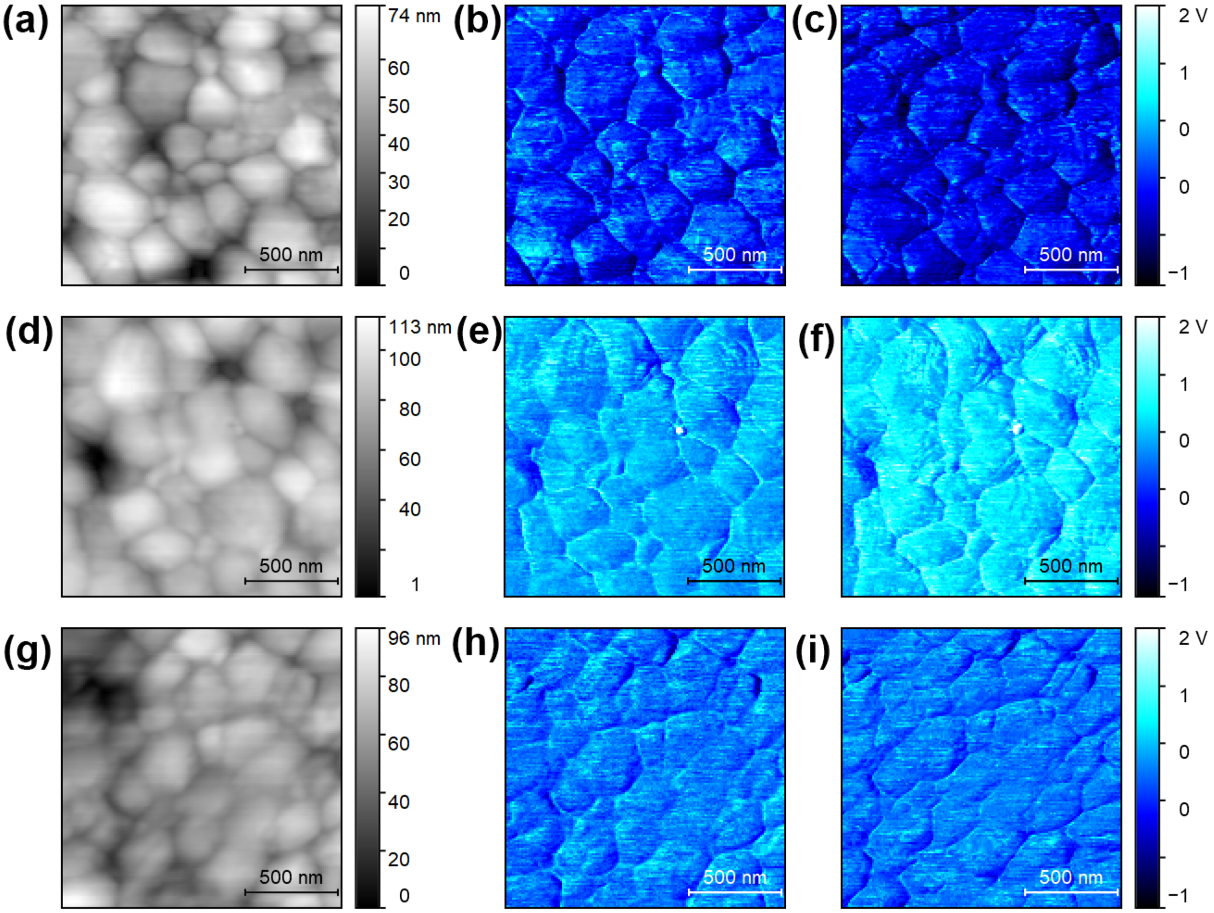

Figure S3. Images from ITO/NiOx NPs/SAMs/DC/PEAI films measured through atomic force microscopy AFM (left) and Kelvin probe force microscopy (KPFM), in dark (middle) and under illumination (right), for 3D perovskite on different SAMs with the partial device stack . 2PACz (a-c), Me-4PACz (d-f) and MeO-2PACz (g-i).

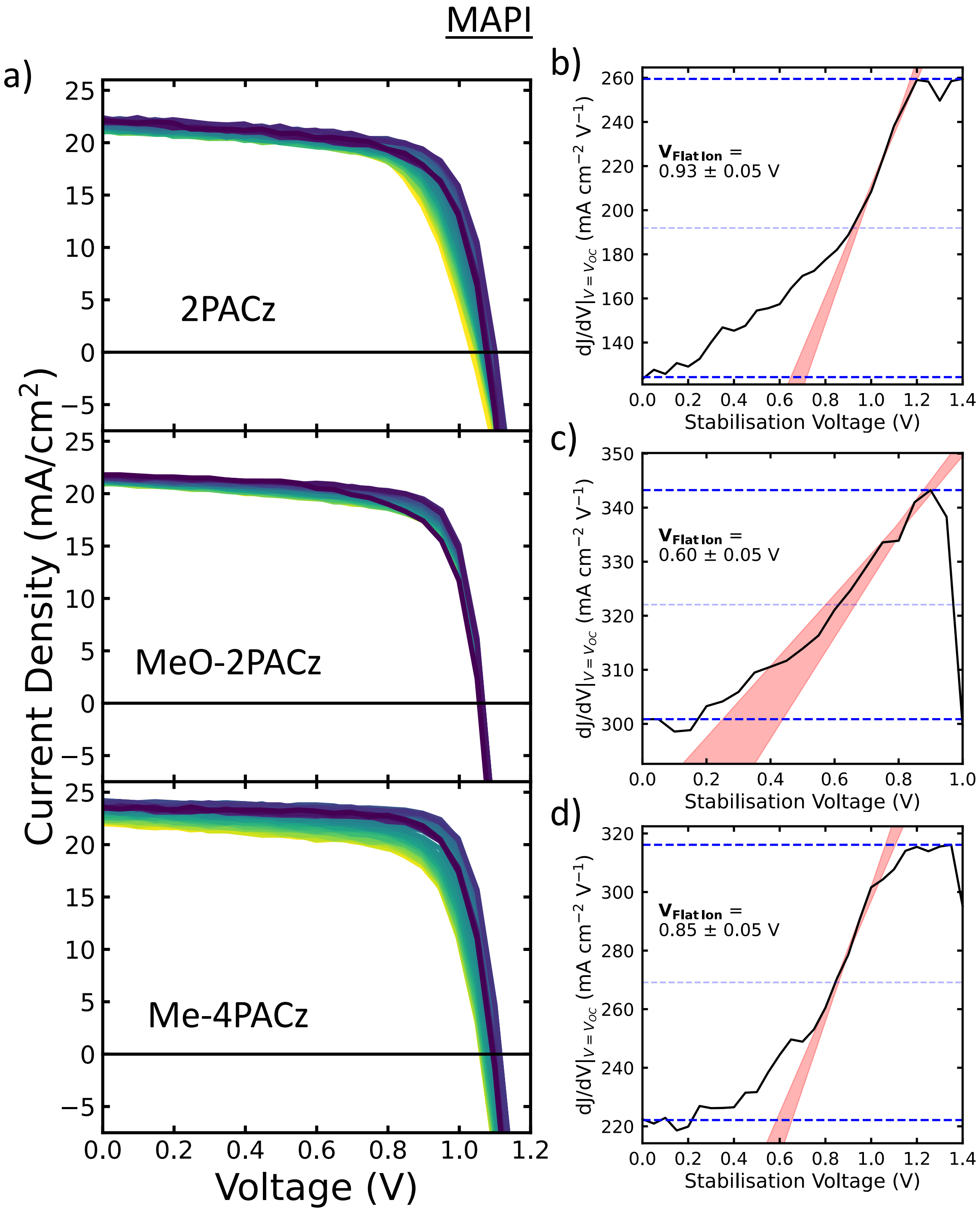
MAPI
a)
2PACz
MeO-2PACz
Me-4PACz
Current Density (mA/cm^2)
Voltage (V)
b)
$V_{Flat\ Ion}$ =
0.93 ± 0.05 V
c)
$V_{Flat\ Ion}$ =
0.60 ± 0.05 V
d)
$V_{Flat\ Ion}$ =
0.85 ± 0.05 V
$dJ/dV|_{V=V_{OC}}$ (mA cm^{-2} V^{-1})
Stabilisation Voltage (V)

Figure S4. Stabilise and Pulse measurements for MAPI perovskite devices using 2PACz, MeO-2PACz and Me-4PACz. a) Full SaP measurement results. dJ/dV analysis around open circuit voltage at each applied bias for devices using b) 2PACz c) MeO-2PACz and d) Me-4PACz.

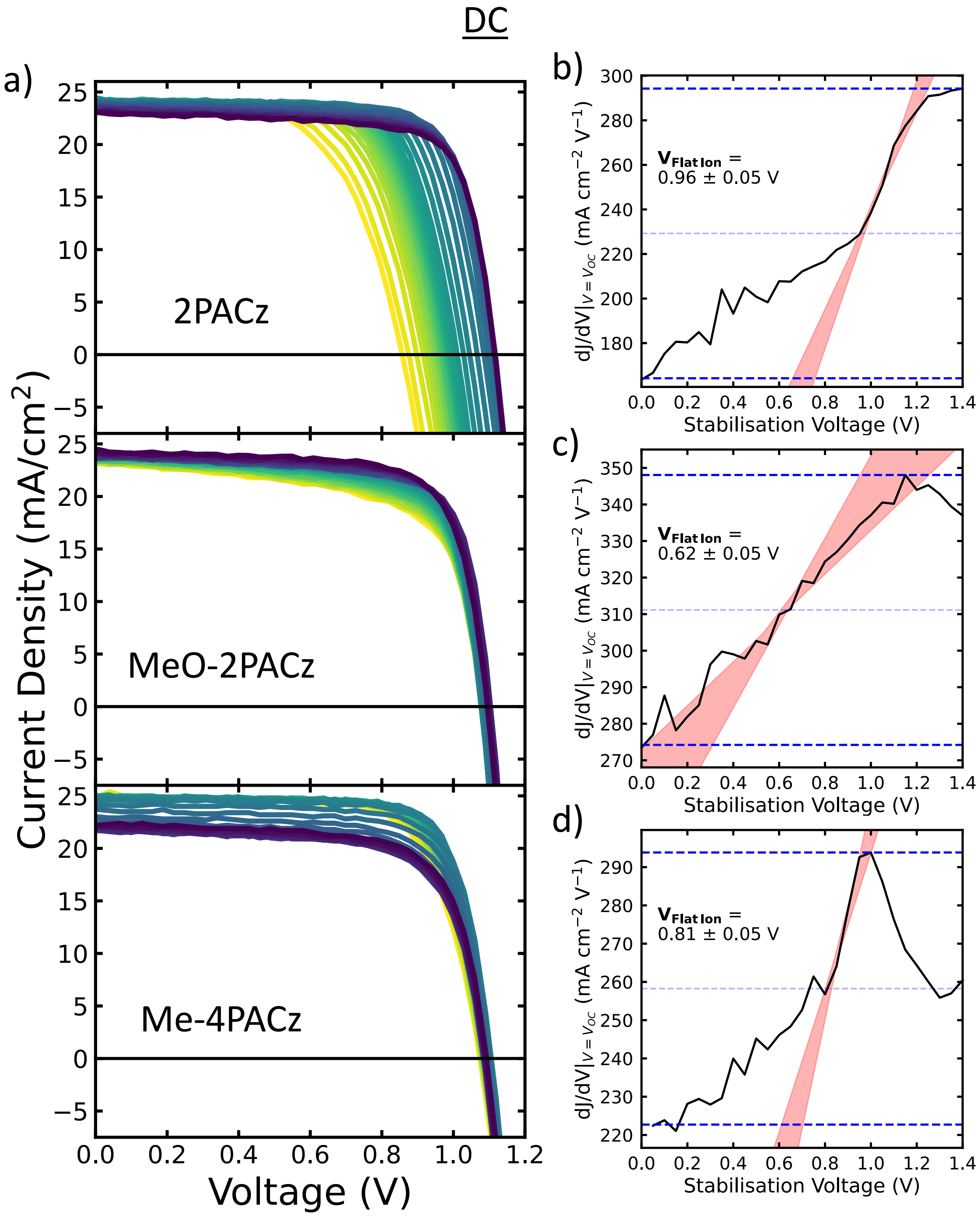

DC
a)
2PACz
MeO-2PACz
Me-4PACz
Current Density (mA/cm²)
Voltage (V)
b)
c)
d)
V_Flat Ion = 0.96 ± 0.05 V
V_Flat Ion = 0.62 ± 0.05 V
V_Flat Ion = 0.81 ± 0.05 V
dJ/dV|V=Voc (mA cm⁻² V⁻¹)
Stabilisation Voltage (V)

Figure S5. Stabilise and Pulse measurements for double cation perovskite devices using 2PACz, MeO-2PACz and Me-4PACz. a) Full SaP measurement results. dJ/dV analysis around open circuit voltage at each applied bias for devices using b) 2PACz c) MeO-2PACz and d) Me-4PACz.

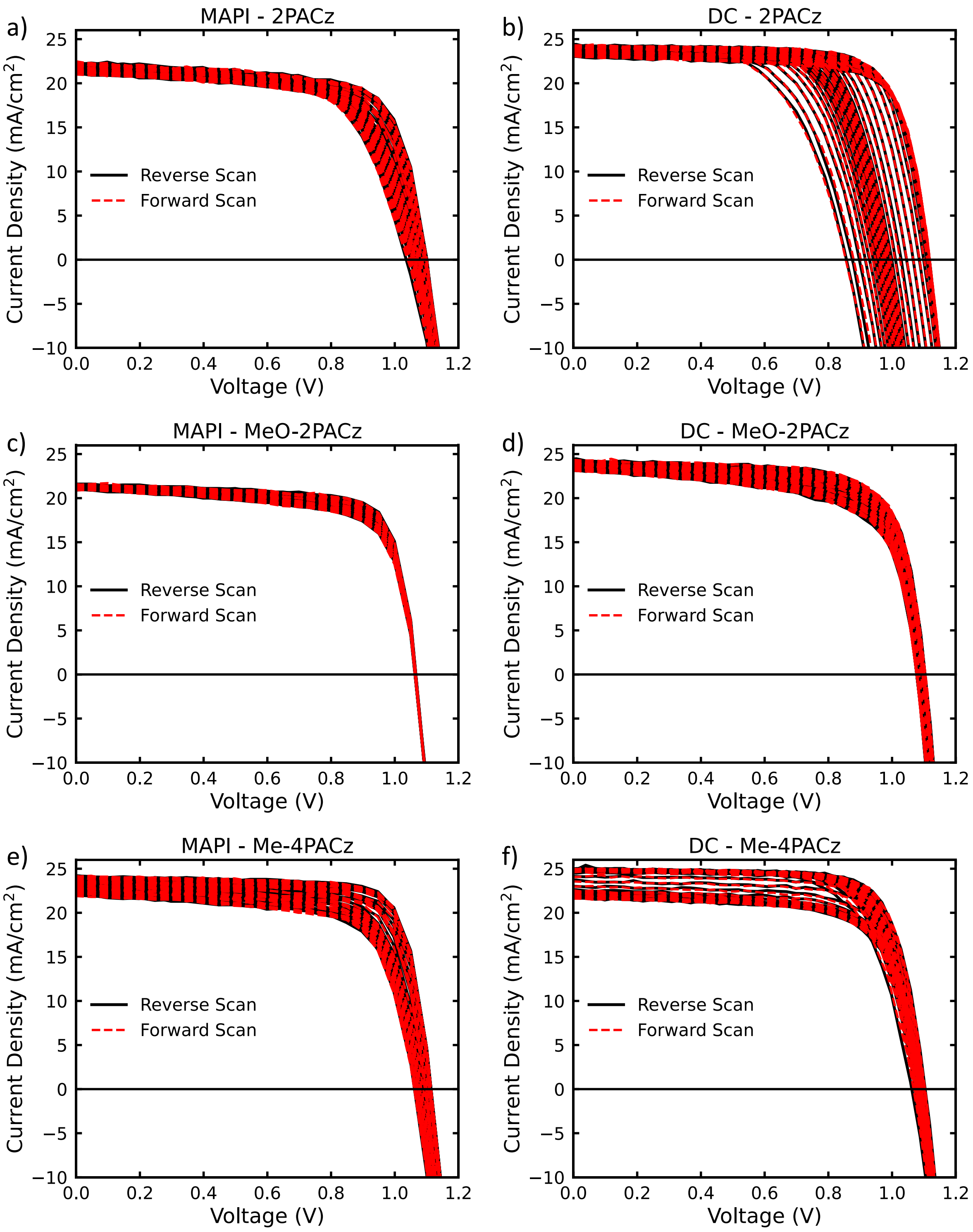

Figure S6. Full reconstructed JV data from the Stabilise and Pulse measurement with contrasting colours to highlight the both scans taken during the measurement. Reverse scans are in black and

forward scans in dashed red for MAPI and DC devices containing a-b) 2PACz c-d) MeO-2PACz e-f) Me-4PACz respectively.

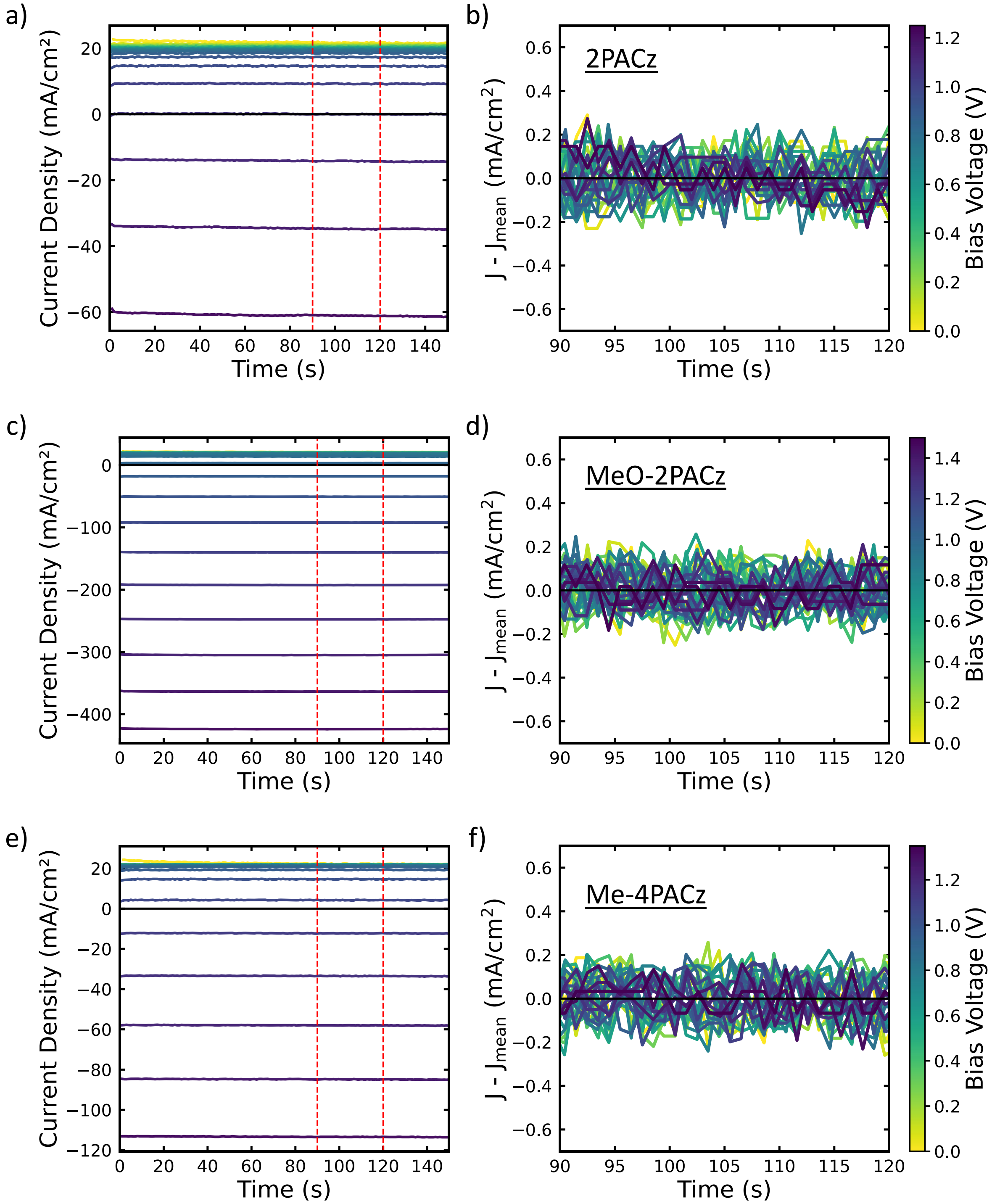

Figure S7. Stabilisation data obtained during Stabilise and Pulse (SaP) measurement for MAPI devices. Figures on the left show the full current density output obtained during each applied bias for a) 2PACz c) MeO-2PACz and e) Me-4PACz. The red dashed lines indicate the final 30 seconds before pulsing for stability analysis. Figures on the right side show the current minus the average current during the final 30s before pulsing beginning for b) 2PACz d) MeO-2PACz and f) Me-4PACz. This shows the ionic configuration was stable before pulsing.

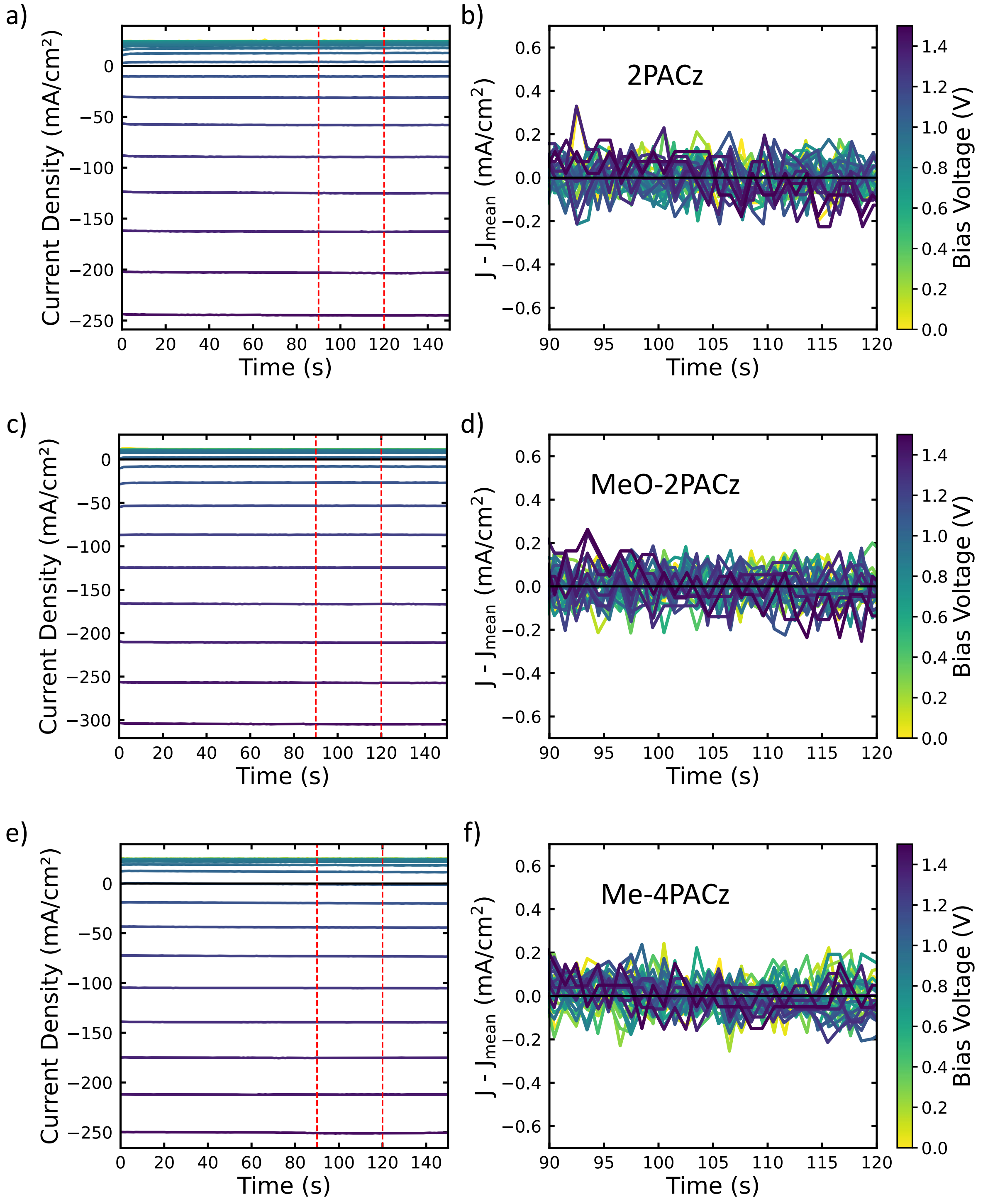

Figure S8. Stabilisation data obtained during Stabilise and Pulse (SaP) measurement for double cation (DC) perovskite devices. Figures on the left show the full current density output obtained during each applied bias for a) 2PACz c) MeO-2PACz and e) Me-4PACz. Figures on the right side

show the current minus the average current during the final 30s before pulsing beginning for b) 2PACz d) MeO-2PACz and f) Me-4PACz. This shows the ionic configuration was stable before pulsing.

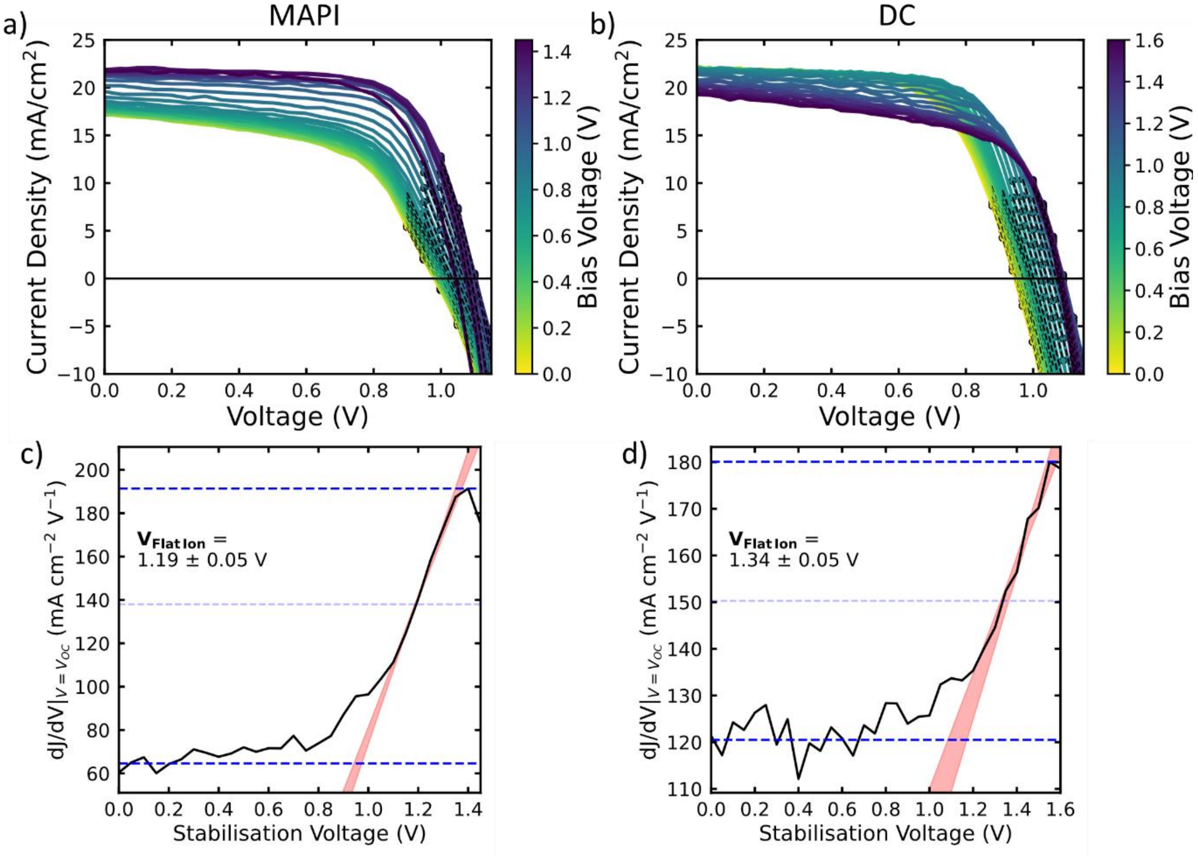

Figure S9. Full reconstructed Stabilise and Pulse measurement of devices utilising the large dipole SAM Cl-2PACz for a) MAPI and b) DC. dJ/dV analysis for devices using the Cl-2PACz for c) MAPI and d) DC. The full stack of the device was ITO|NiOx|Cl-2PACz|Perovskite|PCBM|BCP|Ag, the same as was used for all other SAM-containing devices.

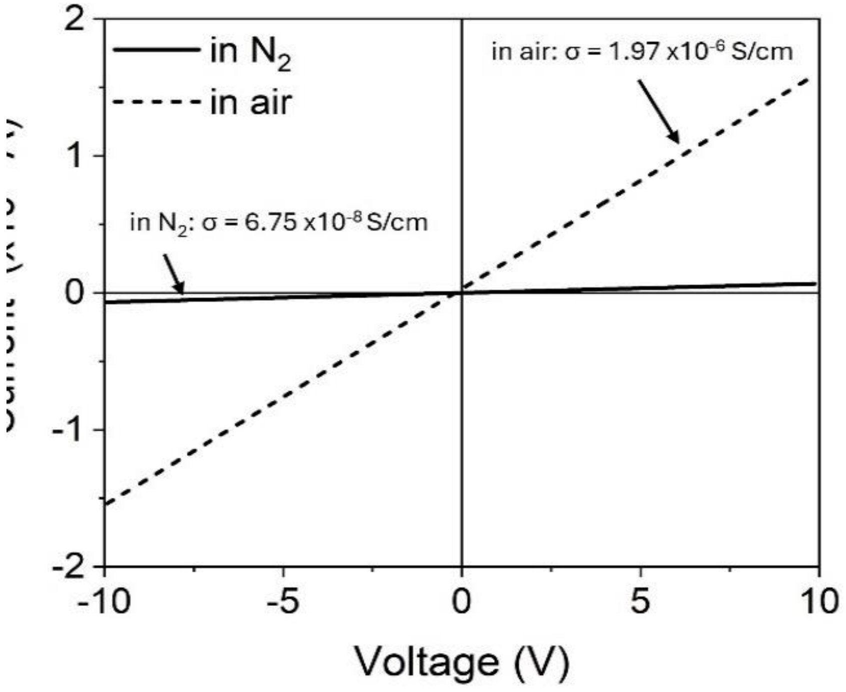

Figure S10. Conductivity measurements of pristine PCBM films exposed in N_2 and air.

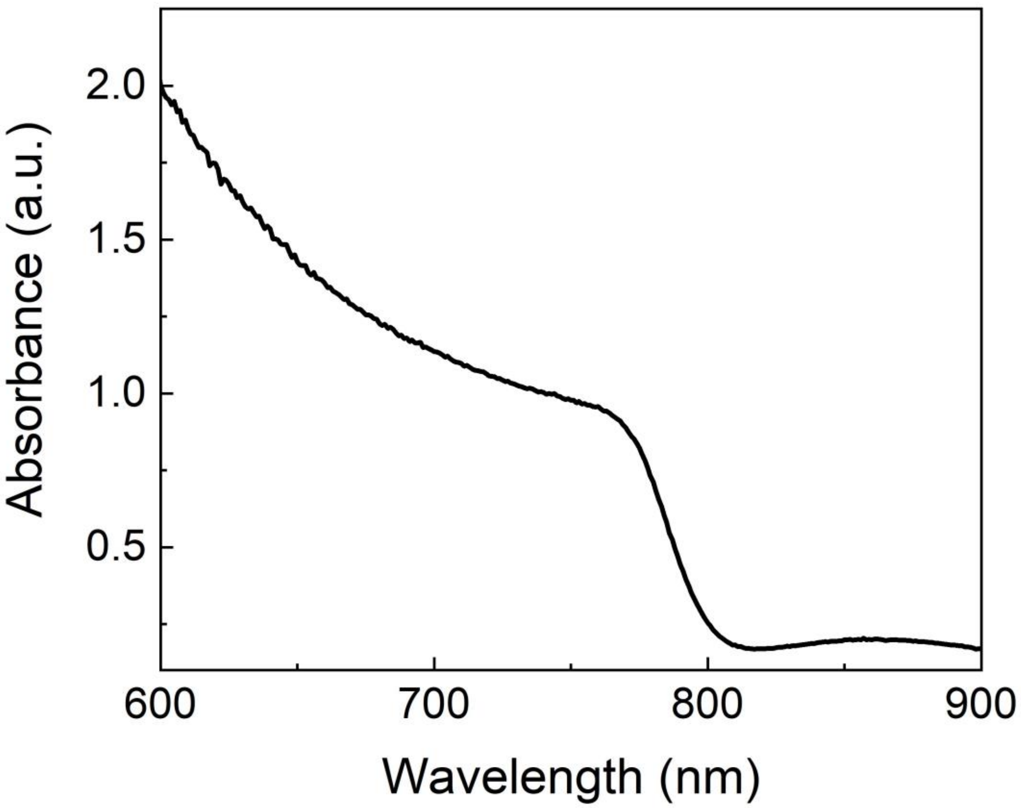

Figure S11. UV-vis spectra of Double Cation perovskite composition.

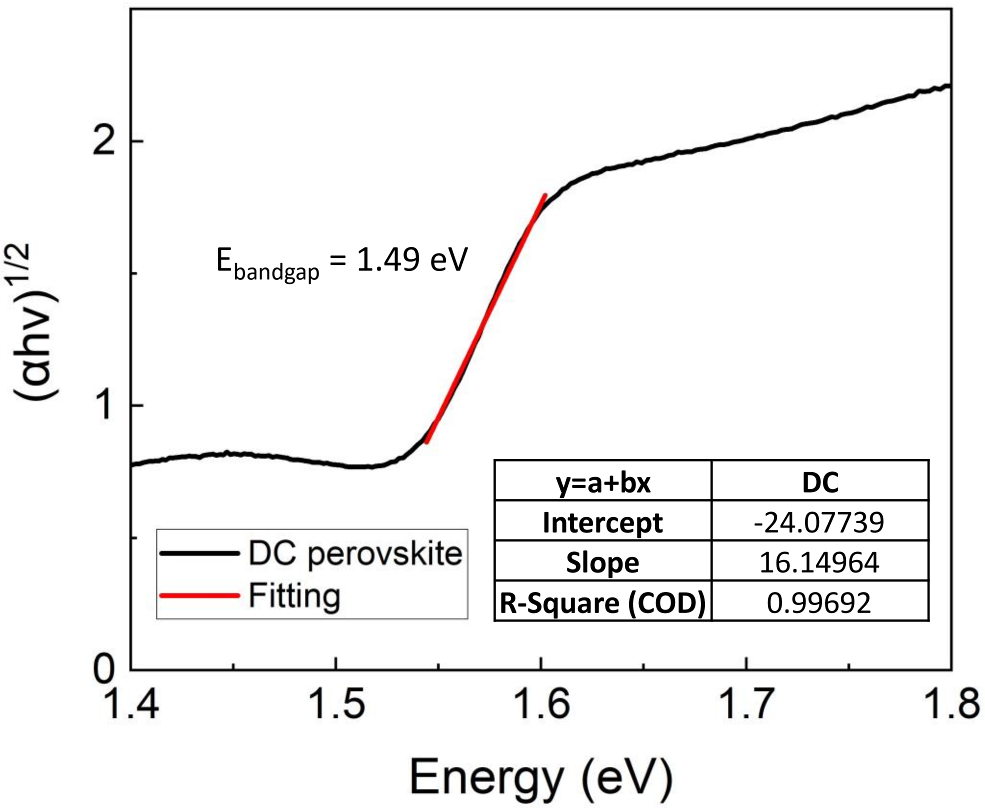

Figure S12. Tauc analysis of double cation perovskite to extract direct bandgap with fitting parameters for band gap extraction as an inset.

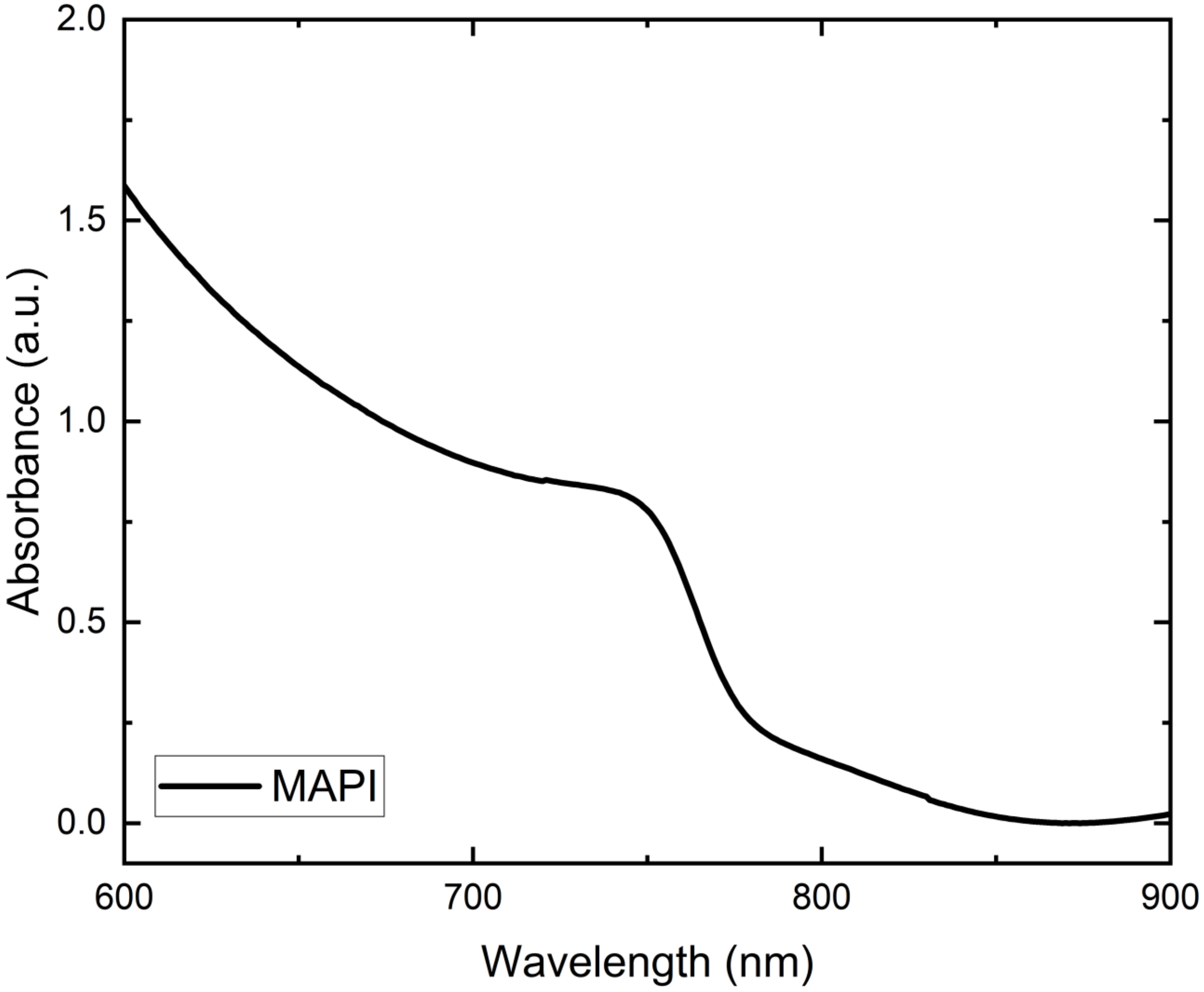

Figure S13. UV-vis spectra of MAPI perovskite composition.

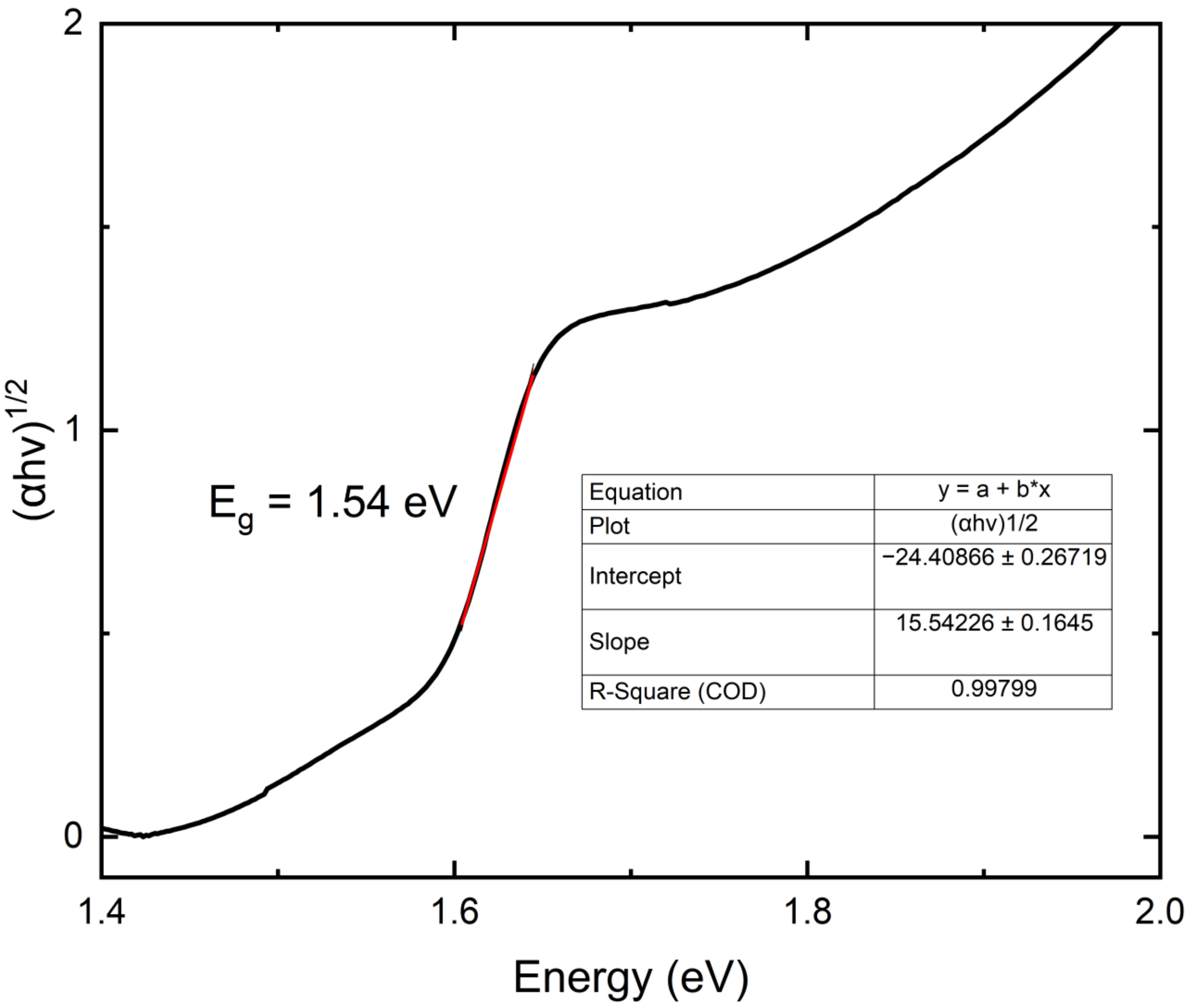

Figure S14. Tauc analysis of MAPI perovskite to extract direct bandgap with fitting parameters for band gap extraction as an inset.

Supplementary Note One

In this supplementary note, we describe the process for extracting the potential drop from the Stabilise and Pulse measurements. To provide an example the MAPI device employing 2PACz is used to show the full analysis. First, the measurement is conducted as described in the **Methods** and the data is processed as shown in **Figure S15a**. As stated in the **Methods** a range of points are obtained following the second-order derivative of the smoothed Savitzky-Golay data being obtained. The Savitzky-Golay filter is used to better represent the obtained data as this method preserves important features and trends in the data while reducing noise, as shown in **Figure S15b**. From this second-order derivative, the inflection point is identified as the maximum value as shown in **Figure S15c**. This point is then used as the central point for analysis and 2 points above and below are used to create a range of data points. This range is then subjected to multiple linear fits through all available options from the obtained data points. The point where the linear fit crosses the middle-value point is the extracted potential drop. This approach was validated with computationally generated data and generally yields a small underestimate of the theoretically inputted value of about 0.05 V for typical perovskite optoelectronic properties.

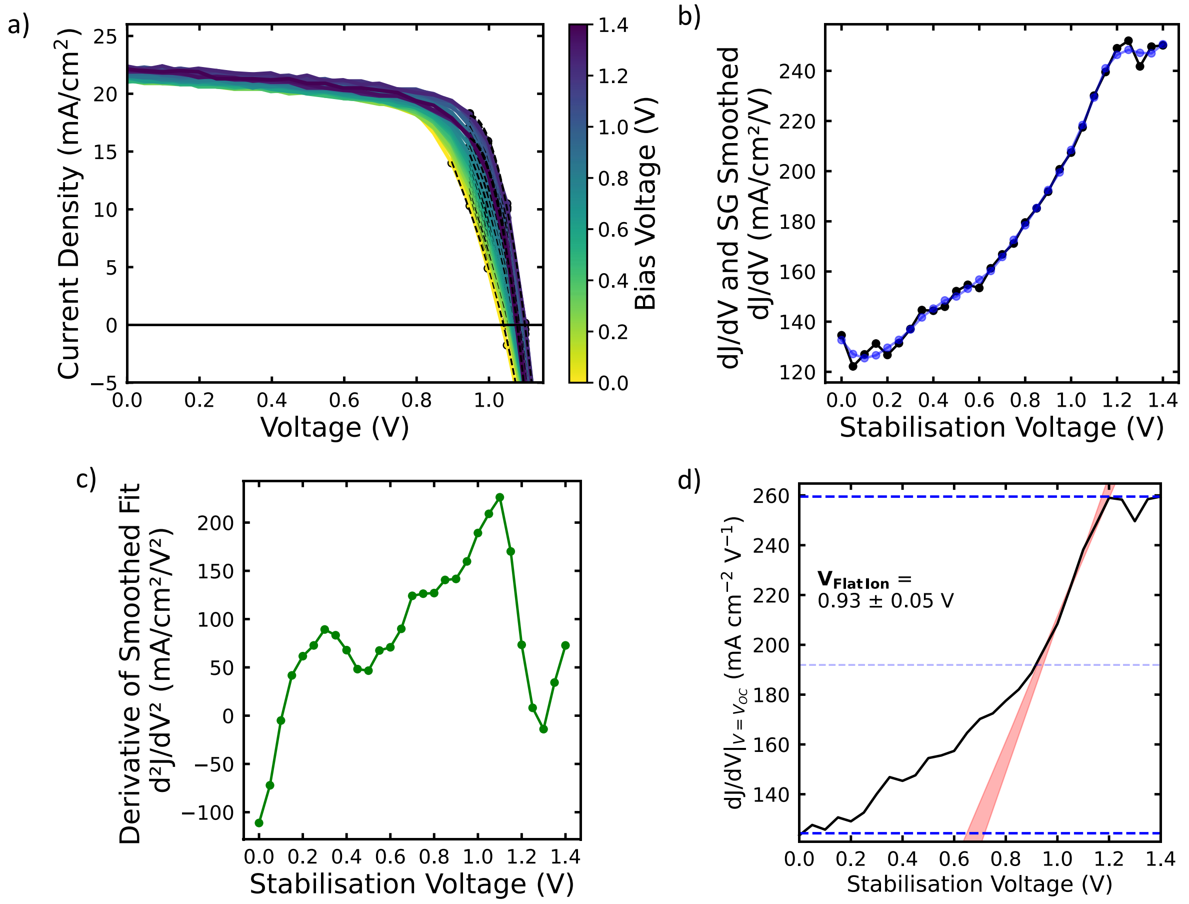

Figure S15. a) Full stabilise and pulse JVs with points used for analysis around Voc shown. b) Gradient around Voc obtained for each applied bias and subsequent Savitzky-Golay 7 point 3rd order polynomial analysis overlapped. c) Second derivative of smoothed data to obtain the inflection point or the steepest section of the curve. d) Final analysis giving calculated electrostatic potential drop across the perovskite layer with the range of linear fits used highlighted within the red shaded area.

In **Figure 15d** we show the range of linear fits used highlighting the maximum and minimum values. To obtain the final value statistical analysis is performed through a weighted average calculation. First, the weighted mean of the linear fits are calculated using **Equation S1**:

$$\bar{x} = \frac{\sum_{i=1}^{N} w_i x_i}{\sum_{i=1}^{N} w_i} \quad \text{(S1)}$$

Where $\bar{x}$ is the weighted average, w_i are the weights and x_i are the observations. We find this allows the data to be better represented rather than selecting a single point and following a singular linear fit. The extracted weighted average is then used as the value and the error is obtained as described in the main text.

References

(1) Johnston, M. B.; Herz, L. M. Hybrid Perovskites for Photovoltaics: Charge-Carrier Recombination, Diffusion, and Radiative Efficiencies. *Acc Chem Res* 2016, *49* (1), 146–154. https://doi.org/10.1021/acs.accounts.5b00411.
