# Supplementary material for: Understanding the Impact of SAM Fermi Levels on High Efficiency p-i-n Perovskite Solar Cells
Source: J Phys Chem Lett. 2024 Oct 16;15(42):10686–95. doi: 10.1021/acs.jpclett.4c02345 (PMC11514011; doi:10.1021/acs.jpclett.4c02345)
Supplement: Supplementary file 2 — jz4c02345_si_002.pdf [file jz4c02345_si_002.pdf]

jz-2024-023452.R1

Name: Peer Review Information for "Understanding the Impact of SAM Fermi Levels on High Efficiency p-i-n Perovskite Solar Cells"

First Round of Reviewer Comments

Reviewer: 1

Comments to the Author

In this manuscript a correlation is made between the potential drop induced in perovskite absorber layers by different SAM hole transporting materials and the device performance. The potential drop is measured by a new method called “Stabilise and Pulse”, where short voltage pulses are used to determine the differential current at a given ionic equilibrium at an interface. This method is necessary in order to differentiate ionic from electronic effect in perovskite materials with high ionic mobility.

It is shown that there is a clear dependence of the potential drop and the solar cell parameters of the device on the SAM dipole. Also, the role of the HOMO-LUMO positions in the SAM layer on the potential drop across the absorber is shown to be much stronger than the electron affinity difference between the two electrodes of the device.

The paper is an important contribution to a better understanding of the interface energetics in perovskite solar cells.

Some comments

- Only three different SAMs are examined in this study. It would be more meaningful with more different materials in order to show that the observed effects can be generalized and are not just coincidental.

- Fig 3: Why is the shape of the dJ/dV curves so different for MAPI vs. DC? Especially the MeO-2PACz curve in the DC case stands out, as it crosses the two other curves due to a much smaller slope. The slope of the three curves in case of MAPI is almost identical for all SAMs, while in the case of DC al slopes are different. Please comment. Also, the legend does not mention the dashed horizontal line in both graphs, this should be corrected.

- On page 15, line 234 is stated: “ This is expected as they both exhibit similar optical bandgaps, see Figures S9 and S10, and, presumably, similar valence and conduction bands as well as Fermi-level positions.” This gives the impression that both band gaps are compared in these figures, however, Figure S9 shows the UV-vis spectrum and Figure S10 the Tauc plot of the DC-

perovskite, while no similar data is shown for MAPI. If this was intended, please change the sentence above.

Author's Response to Peer Review Comments:

Dear Editor,

Thank you very much for this opportunity to amend our submitted article. We have now addressed all the comments raised by the referee, where our point by point response is attached in the 'response to referee comments' document.

In addition to the comments and corrections made we took this opportunity to improve the text for clarity/legibility. We have additionally updated the TRPL result section to incorporate more meaningful fits extracted from a bimolecular-trapping-auger recombination model. We note that these results do not change the interpretation of the data, but are simply more representative than the bi-exponential model used previously. These changes are now highlighted in the submitted manuscript file for completeness.

We look forward to hearing back from you.

Best regards,

Dr Pablo Docampo

Response to Reviewers' Comments on, 'Understanding the Impact of SAM Fermi Levels on High Efficiency p-i-n Perovskite Solar Cells'

Reviewers' comments are bolded in black, the author's responses are in blue and quotations from the paper are italicised.

Reviewer One

Our response:

We are grateful to the reviewer for their thoughtful comments and thorough consideration of our work. Our responses to the comments are included below.

Comment 1: Only three different SAMs are examined in this study. It would be more meaningful with more different materials in order to show that the observed effects can be generalized and are not just coincidental.

Our response:

We thank the reviewer for suggesting an additional material could be incorporated into this work and agree it would increase the impact. A good SAM candidate to verify the trends hold in more extreme cases is provided by Cl-2PACz, first introduced by Lin et al. (Y. Lin, Y. Zhang, J. Zhang, M. Marcinskas, T. Malinauskas, A. Magomedov, M. I. Nugraha, D. Kaltsas, D. R. Naphade, G. T. Harrison, A. El-Labban, S. Barlow, S. De Wolf, E. Wang, I. McCulloch, L. Tsetseris, V. Getautis, S. R. Marder, T. D. Anthopoulos, 18.9% Efficient Organic Solar Cells Based on n-Doped Bulk-Heterojunction and Halogen-Substituted Self-Assembled Monolayers as Hole Extracting Interlayers. *Adv. Energy Mater.* 2022, 12, 2202503.)

This SAM layer includes the extreme case with a strong dipole moment of ~4.6 D as determined by Wang et al. (Y. Wang, W. Jiang, S.-C. Liu, C.-T. Lin, B. Fan, Y. Li, H. Gao, M. Liu, F. R. Lin, A. K. Jen, Durable Organic Photovoltaics Enabled by a Morphology-Stabilizing Hole-Selective Self-Assembled Monolayer. *Adv. Energy Mater.* 2024, 14, 2303354.) As requested, we have thus fabricated solar cells incorporating this material and studied the resulting behaviour using the established Stabilise and Pulse methodology.

We find that for both MAPI and DC devices the Flat ion potential increases significantly, where we observe a value of approximately ~ 1.2 V for MAPI and ~ 1.3 V for DC when the high dipole Cl-2PACz is used. This aligns with our previous results that showed a rise in Flat ion potential alongside increasing dipole moment. We have added this data to the supplementary information and adjusted the main text as follows, from line 246:

"A summary of the extracted values is presented in Table 1. Our results show a clear trend of the potential drops across the perovskite layer that correlates with the strength of the dipole moment of the underlying SAM layer. We confirm this trend by using a chlorine-substituted carbazole SAM, Cl-2PACz, with a dipole moment of ~4.6 D41,42, much greater than that of the other materials utilised. With the large dipole induced by Cl-2PACz, we extract a Vflat of > 1.20 V for both MAPI and DC devices. This result confirms the correlation between a large Vflat with increased dipole strength. The full SaP and dJ/dV analysis for these devices can be seen in Supplementary Figure S9."

Supplementary Figure S9:

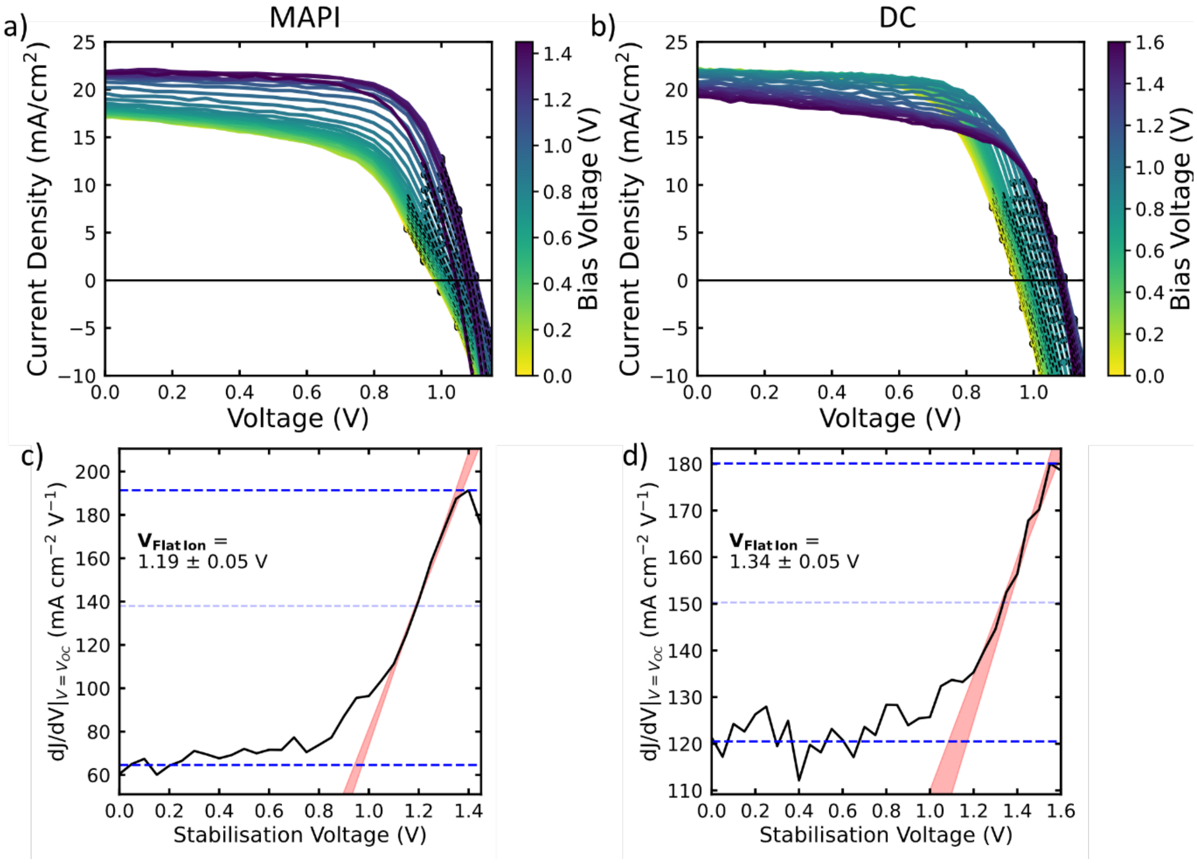

Figure S9. Full Stabilise and Pulse curves for devices containing Cl-2PACz for both a) MAPI and b) DC. dJ/dV analysis of Cl-2PACz devices on c) MAPI and d) DC. The full stack of the device was ITO|NiOx|Cl-2PACz|Perovskite|PCBM|BCP|Ag, the same as was used for all other SAM-containing devices.

Comment 2: Fig 3: Why is the shape of the dJ/dV curves so different for MAPI vs. DC? Especially the MeO-2PACz curve in the DC case stands out, as it crosses the two other curves due to a much smaller slope. The slope of the three curves in case of MAPI is almost identical for all SAMs, while in the case of DC al slopes are different.

Our response:

We thank the reviewer for highlighting that further discussion regarding the slope of the dJ/dV curves in this paper is necessary. The dJ/dV analysis extracted from the SaP measurements quantifies the effect of ion accumulation at the interfaces on charge extraction and recombination. It is important to note here that the slope of this curve reflects the balance between ion-modulated charge recombination at the interface and losses within the bulk. If bulk recombination increases, then the interface is 'less dominant' and thus dJ/dV will vary at a lower rate with applied bias, i.e. ion accumulation at the interface has less effect on the shape of the JV curve and consequently the slope of the dJ/dV curve becomes less pronounced. In systems with a high density of very mobile ions such as MAPI, the shape of the JV curve is dominated by interfacial recombination which is driven by the accumulation of these ionic species. This results in the well-known and very visible JV hysteresis for the vast majority of MAPI-based solar cells regardless of contact materials used. DC systems are well known to contain a far lower number of ionic species that also diffuse more slowly. In combination, this reduces interfacial recombination, reducing JV hysteresis and consequently reducing the slope of the curves produced through the dJ/dV analysis. This observation is consistent with our results -- we have postulated that the lower built-in potential present in devices incorporating MeO-2PACz induces a higher degree of bulk recombination compared to the other two SAM layers studied due to the bigger overlap of the electron and hole populations. The fact that the dJ/dV analysis curve for MeO-2PACz has a lower slope than the other two systems gives further evidence that our hypothesis is correct. We have modified the main text to incorporate this important point in the following manner, from line 268:

"The dJ/dV analysis, derived from the SaP measurements, provides insights into the effects of ion accumulation at interfaces on charge extraction and recombination. The slope of the dJ/dV curve reflects the interplay between ion-modulated interfacial charge recombination and bulk losses. As bulk recombination increases, the interface becomes less dominant, causing a less pronounced slope in the dJ/dV curve. In systems with high densities of mobile ions, such as MAPI, interfacial recombination dominates due to the large density of mobile ions, which is typically in the range of 10^{17} to 10^{19} cm^{-3}.[45–48] This results in the well-known JV hysteresis observed in most MAPI-based solar cells regardless of the contact materials used. On the other hand, DC perovskites, with fewer and slower-diffusing ionic species,45 exhibit reduced interfacial recombination and JV hysteresis and therefore are expected to show a shallower slope in the dJ/dV analysis.

This is reflected in the results obtained as the extracted data shows that the dJ/dV fits to the MAPI devices are more accurate, while the DC perovskite produces a noisier response. Further to this, we note that the DC device incorporating MeO-2PACz has a less steep slope for dJ/dV, where its trace crosses over the other devices measured. This shallow slope is indicative of reduced ionic impact and, therefore, an increased bulk-to-surface recombination ratio. This is in agreement with our analysis of the TRPL and KPFM data which indicated an increased overlap of photogenerated charge."

Comment 3: Also, the legend does not mention the dashed horizontal line in both graphs, this should be corrected.

Our response:

We thank the reviewer for highlighting this mistake and have now rectified it:

*"**Figure 3.** Normalised dJ/dV analysis of devices using all SAM layers for a) MAPI and b) DC. 2PACz is in black, Me-4PACz in red and MeO-2PACz in blue. As a guide to the eye, we include a Savitzky-Golay smoothed line in a lighter shade. The dashed line represents the best linear fit to the steepest part of each curve. The grey horizontal line represents the halfway point of the data and is used in combination with the linear fit to extract the flat ion potential (V_{flat}). For more information see **Supplementary Note 1**."*

Comment 4: On page 15, line 234 is stated: " This is expected as they both exhibit similar optical bandgaps, see Figures S9 and S10, and, presumably, similar valence and conduction bands as well as Fermi-level positions." This gives the impression that both band gaps are compared in these figures, however, Figure S9 shows the UV-vis spectrum and Figure S10 the Tauc plot of the DC-perovskite, while no similar data is shown for MAPI. If this was intended, please change the sentence above.

Our response:

We thank the reviewer for identifying this error. We have corrected this by incorporating the absorption data for MAPI and amending the text as follows:

Deleted: This is expected as they both exhibit similar optical bandgaps,63 see Figures S9 and S10, and, presumably, similar valence and conduction bands as well as Fermi-level positions.

Replaced:

*"We also note the neat agreement between the values obtained for the different perovskite compositions utilising the same SAM layers. This is expected since both perovskite compositions exhibit similar band gaps,[27] as can be seen from our absorption and Tauc analysis of both the DC perovskite in **Figures S11 and S12** and for MAPI in **Figures S13 and S14**. From this, it is reasonable to assume that they will exhibit similar valence and conduction band positions as well as Fermi level. Furthermore, taking both sets of results together, we can be confident that the value of the V_{flat} extracted here is therefore solely a result of the difference in Fermi levels induced by the different SAMs anchored on the metal oxide layer."*

Supplementary Figures S13 and S14:

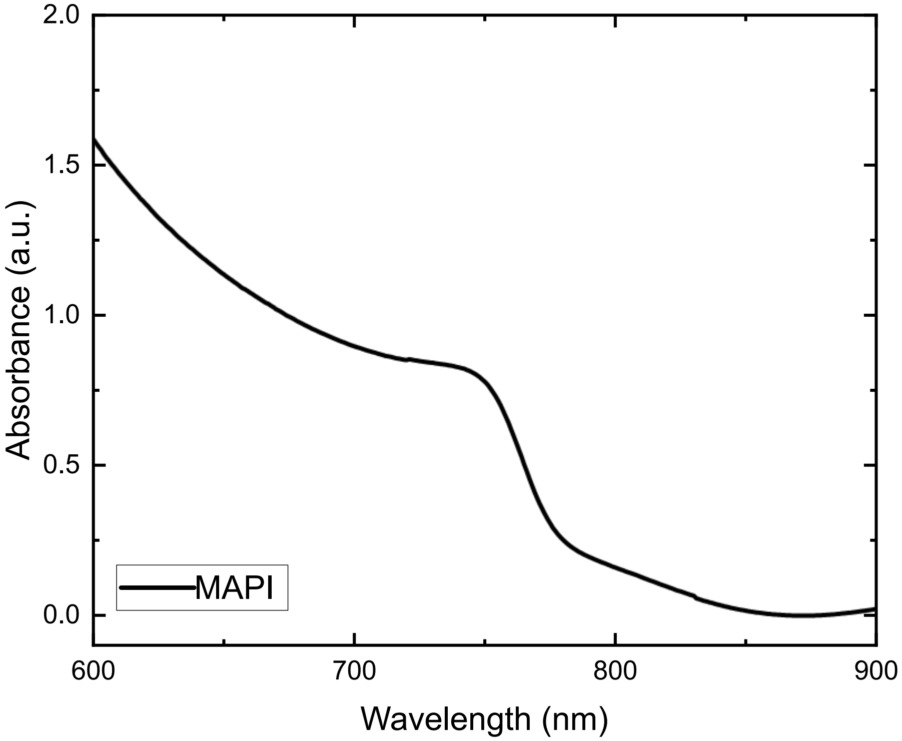

Figure S13. UV-vis spectrum of MAPI perovskite composition.

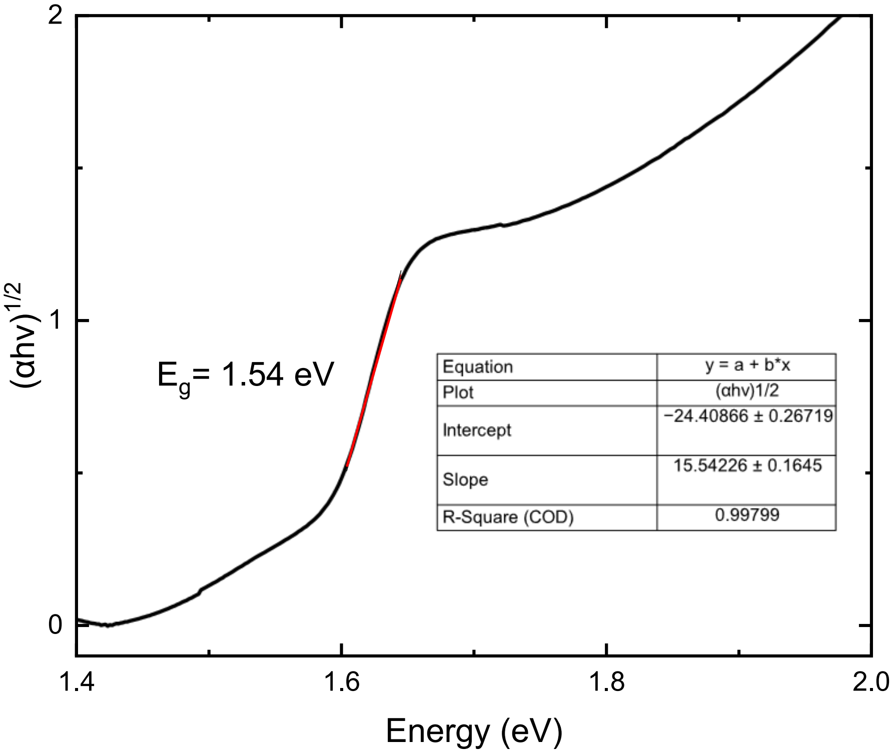

Figure S14. Tauc analysis of MAPI perovskite to extract direct bandgap with fitting parameters for band gap extraction as an inset.

Additional amendments:

Updated citation due to article being published:

Was - (33) Hart, L. J. F.; Angus, F. J.; Li, Y.; Khaleed, A.; Durrant, J. R.; Djurišić, A.; Docampo, P.; Barnes, P. R. F. More Is Different: Mobile Ions Improve the Design Tolerances of Perovskite Solar Cells. **2024**. arXiv:2407.04523 [cond-mat.mtrl-sci]

Now - (27) Hart, L. J. F.; Angus, F. J.; Li, Y.; Khaleed, A.; Calado, P.; Durrant, J. R.; Djurišić, A. B.; Docampo, P.; Barnes, P. R. F. More Is Different: Mobile Ions Improve the Design Tolerances of Perovskite Solar Cells. *Energy Environ Sci* **2024**. https://doi.org/10.1039/D4EE02669A.
